# Spirit of the English Periodicals, and Notices of English Medical Literature

**Published:** 1834-07-01

**Authors:** 


### Periscope;

OR,

### CIRCUMSPECTIVE REVIEW.

" Ore trahit quodcunque potest, atque addit acervo."

I.

### Spirit of the English Periodicals, and Notices of English Medical Literature.

A NEW SYNOPSIS OF NOSOLOGY, &c. By Dr. G. H. WEATHERHEAD. Small Octavo, 1834.

WE have been informed on good authority, that Dr. Weatherhead spent many hours of the day for several years in constructing this system of nosology. We hope the time was spent as pleasantly and profitably as the hours, days, and months passed in traversing France, Italy, and Switzerland, with his knapsack on his back, pursuing his " Pedestrian Tour." We candidly confess our opinion, that in these nosological labours of Dr. W. the workmanship surpasses the work, and that the labourer, though worthy of his hire, will be little likely to receive his reward. The series of nosological systems, from Plater to John Mason Good, each one pointing out the errors and absurdities of its predecessors, shews the unstable basis on which nosology rests. It was all very well for our first parent to classify and name the various animals that passed before him : but it is a very different matter for the physician to classify and arrange diseases that change their forms, symptoms, and even their nature, as rapidly as Proteus. Inflammation to day, is often dropsy to-morrow—and Arthritis in the morning may take the name of Pericarditis in the evening! Dr. Good laughed at Dr. Cullen for arranging itch and broken bones together —while Dr. Weatherhead has his fling at Good for classing toothache with prolapsus ani. We do not think it would be very difficult to point out some incongruities even in Dr. W.'s nosology. Thus, in the classification of catalepsy with varicose veins, we confess that we have puzzled our imaginations a good deal to discover the link by which these two diseases are connected. Many such strange *associations* might be pointed out, and some not less curious *dissociations*. Thus neuralgia is designated as *idiopathic*, when the seat of pain is in the face or heel; but *sympathetic*, when in the nerves spread over the pericranium. Nevertheless we believe that the principle on which our author constructs his system is as good, if not better than that which guided either Cullen or Good.

" I hope to make obvious, in the sequel, the propriety, as well as utility, of founding nosology on the properties of the tissues, as far as our knowledge of them goes : the similarity of their several symptoms when diseased, the resemblance in their results or effects, and, above all, the sameness of the principle requisite to be pursued in their treatment, in whatever part of the body the tissue may be situated, all demonstrate the advantages derivable from a classification taking this principle for its foundation.

On referring to systematic, and other writers, on diseases, we have recurring in almost every page occasion for surprise at the Protean form that the symptoms of the same disease are described as assuming—now present, now absent, sometimes acute, at other times obtuse; and if we come to examine how this happens, we shall find that it is attributable, in amost every instance, to the morbid action not being seated in the same tissue. This is more particularly observable in the diseases of compound organs; but in all these cases, on properly locating the seat of the morbid action, all contrariety and contradiction in the symptomatology is god rid of at once. It is true the morbid action set up in one tissue will, and does frequently, extend to a different tissue immediately contiguous, and then the symptoms peculiar to each become mixed; but, even in this case, a knowledge of these peculiarities assists rather than confounds the correctness of our judgment as to the nature and extent of the disease; for it will always be found, that the assemblage of symptoms present was not of simultaneous appearance, but that a part of them was superinduced on the other; thus shewing the progression of the diseased action by extension or sympathy."

Dr. W. has classified the diseases of the human body under four principal heads. These form his classes—namely, the PHLOGOTICI, or pure inflammatory diseases—the HÆMAPHARMATICI, or those diseases which originate from a miasm or poison entering the blood, and tainting it—thirdly, the NEUROTICI, or diseases of the nervous system—and lastly, VITIA, comprising all accidental disfigurations, new morbid formations, extraneous lodgments, and congenital malformations. Upon each of these classes Dr. W. makes many pertinent observations—more especially on the *sequelæ* of diseases, in arranging which, nosologists have not drawn the proper distinction between them and their primary causes. Each of these sequelæ or results will find a place (Dr. W. thinks) in this new synopsis.

"But it may be well to elucidate my meaning more distinctly, by exemplifying it. Let us take subacute hepatitis as the primary disease: a common morbid consequence of this is the interstitial deposition of coagulable lymph into its structure, which, on becoming organised, constitutes scirrhus of the liver. This is a sequela of the primary morbid action, but it is not its ultimate result, as we shall see by pursuing the subject a little further. Scirrhosity gives a preternatural solidity and induration to the liver: this naturally presents a constant and powerful obstruction to the free return of the venous blood from several of the abdominal viscera; a mechanical hæmostasis or congestion of necessity takes place in all the branches of the vena porta, which, in order to relieve themselves of the engorgement, force into the exhalants opening into the cavity of the abdomen an undue portion of serosity greater than the absorbents can remove, the morbid consequence of which is ascites. Here, then, we have two consecutive morbid sequelæ, each constituting a specific disease, the one being the subsequelæ of the other, besides the primary disease; indeed, it not unfrequently happens, that all these three morbid states shall simultaneously coexist. The following, therefore presents an illustrative diagram of the subsequent synoptical arrangements:—

*Species* 1. HEPATITIS.
*Variety*. a. Sub-acuta.
*Sequela*. *a*. Scirrhus.
*Subsequelæ*. a. Ascites.
b. Hæmorrhœa intestinalis.
*a*. Recti.

The reader will further perceive b. *Hæmorrhœa intestinalis* and a. *Recti* superadded, by which it is intended to indicate, that sometimes, instead of dropsy being the sequela, as explained above, intestinal hæmorrhœa shall be the morbid consequence of the sanguineous engorgement; and the word "Recti" is placed to shew when this proceeds from a particular part of the intestines, that is, from the hæmorrhoidal veins."

We acknowledge that this plan, if it could be followed out correctly in all its details, would be much more natural and practically useful than those artificial systems hitherto adopted by nosologists. But immense difficulties are strewed in the path of the nosographer, in our present state of knowledge, since the succession of diseases is very obscurely marked by their symptoms or external phenomena.

Nosology may be considered the grammar of medicine. It is useful to the teacher and to the pupil, as facilitating the communication and the reception of medical knowledge. When the practitioner has launched his bark for a few years, he loses sight of his nosology, in a great measure—or rather, he forms a practical one of his own, by which he steers through the quicksands and straits of his professional voyage of life. To our medical grammarians or schoolmasters, now so numerous a race in the world, we recommend Dr. W.'s nosology; for by them, rather than by the practical physician, immersed in the observation and treatment of diseases, must its merits be decided.

---

### Anomalous Mental Affection.

In a recent number of the Lancet Mr. Lambert has detailed a curious case of which we shall here condense the chief features. The subject was an intelligent girl, about fifteen years of age, who while residing in the country, with some relations, in the absence of her mother, was either ill treated, or fancied herself so. The mother having settled in London, sent for her daughter, but the relations above-mentioned endeavoured to dissuade her from returning, alarming her with the fears of maltreatment from her step-father. On returning, however, she was agreeably disappointed, by experiencing the kindest reception. Yet this transition from negligence to parental affection seemed to fix more deeply her melancholy and abstraction. She endeavoured to keep from society, and wept alone. Five or six months passed thus, with a perceptible increase of the mental malady. Leeches were applied to the temples, and drastic purgatives were exhibited, with some advantage; but she soon relapsed, and complained of violent pains in the head. She was alternately elevated and depressed in excess, using extravagant language and gesticulations, without any adequate cause. A physician in Surrey, and afterwards two other medical men prescribed for, and were completely puzzled by the anomalies of the case. After the application of a blister, she had a succession of fainting fits—and after that, she appeared to lose the sense of feeling over the body. "A slap on the naked shoulder, the palm of the hand, the cheek, or even a pinch on any sensible part, were all equally disregarded." Her sense of hearing was also greatly obtunded, and vision was obscured. In fine, there is no end to the extravagancies which she exhibits, and it would be useless as well as tedious to relate them here. It may be observed however, that the girl maintains the appearance of robust health, though she does "not consume one ounce of food in the day." Bread and cheese are her favourite food, and even that but sparingly and seldom. She drinks but little, and that porter. She has not yet menstruated.

*Remarks.* Although no two of these cases are precisely similar, yet we have seen several of the same nature as the above. It is hysteria, and we have no doubt that the girl will recover, though the period is uncertain. We would recommend leeching the groins or the labia, with the hip-bath and such means as are likely to bring on menstruation.

---

### Fatal Effusion of Blood into the Pericardium. By Dr. Carson, of Liverpool.

The following very interesting case we copy from the first number of the Liverpool Medical Journal—a new contemporary, which augurs well, if we may judge by the first specimen.

"Mr. W. a gentleman about 52 years of age, of a tall and robust form, clear complexion, subject occasionally to dyspeptic affections; though of very regular and temperate habits; of an active disposition, though his occupation was sedentary and confining; had been for twelve months affected with considerable anxiety of mind, in consequence of the doubtful issue of some building speculations. Towards the end of Lent, which he had rigidly observed according to the injunctions of the Catholic Church, on the 11th of March, a day exempted from the prohibitions respecting diet, he had eaten freely of beef-steaks with onion sauce. He was at that meal sparing as usual in the use of wine. On the evening of the following day, he was engaged in a fatiguing and rather anxious way, with the business of a club, of which he was treasurer. On his return from the club, about eleven o'clock at night, in company with two of his friends, when he had nearly reached his own house, he was seized with faintness and debility to such a degree, that without the assistance of the friends who accompanied him he would not have been able to have kept his feet. Soon after his arrival at his house, he was visited by Mr. Bromilow, his medical attendant. He described himself as faint and exhausted; complained of an obtuse, heavy pain at the precordia, and was affected with flatulent eructations. His respiration was free, his pulse 70, and regular, though weak. He had no affection of the head, nor pain any where, excepting as described in the chest. His bowels had been opened that day. Mr. Bromilow ordered an antispasmodic draught; and left him with directions to take something warm, and go to bed. He took the draught, and a weak glass of brandy and water. At three o'clock he sent for Mr. B. again, and, as the pain in the chest was not abated, he expressed a wish to be bled, which Mr. B. agreed to, more with the hope of satisfying his mind than from any great necessity for that measure being indicated by the symptoms. He lost a pint of blood. An opiate was then administered. At this visit, Mr. B. examined the chest more minutely. He applied his ear to the different regions of the naked chest, but perceiving no unusual sound, or vibrations, concluded that the heart, lungs, and large vessels were in a sound state. At five o'clock, a. m. I visited him. He felt cold, perspired gently, and chiefly complained of a pain in the chest, which he described as wearisome and oppressive. It was not increased by taking a full inspiration. He had vomited a little in the course of the night, and had discharged some of the onion sauce he had taken the day preceding the attack. He was much troubled with flatulency, and belched frequently, but was not relieved by it so far as regarded the pain in the chest. His pulse was regular; the heat of the body natural; and respiration good. He had had no sleep.

From the information given by Mr. Bromilow, connected with my own observation, I considered that nothing could be indicated by the symptoms beyond an affection of the stomach, which is known to exhibit itself in such anomalous forms. He took four grains of calomel, and two of opium. We visited him again at half after eleven o'clock. He had had little sleep. The symptoms remained the same. He was ordered an aperient mixture, and we proposed to visit him again at seven o'clock. At this visit, I replied to the anxious inquiries of the family—that we did not see any cause for alarm; that the complaint seemed to arise from indigestion; and that I had no doubt he would recover. At three o'clock in the afternoon he sent for Mr. Bromilow, as the pain still continued unabated, and wished to know if he might have any thing to rub the part with. The bowels had not been opened, and he had had little or no sleep. A short time before seven o'clock, the hour at which we had proposed to visit him, and at which I was prevented from attendance by an urgent call to a distant part of the country, Mr. W. was seized with what the family conceived to be a fit; and a short time after the arrival of Mr. Bromilow, expired. In consequence of my unavoidable absence, other physicians were called in, and two arrived, but not until after the death of the patient. I applied for permission to open the body, which was granted. The body was examined twenty-four hours after death, by Mr. Bromilow in my presence, and in that of my son, Dr. Carson, jun. The following were the appearances on dissection. Upon opening the chest, the lungs on both sides were perfectly sound and collapsed. But, notwithstanding the collapse, the chest was filled more than it usually is when the lungs are sound. This indicated the existence of some foreign substance or morbid enlargement of some of the organs. The pericardium was found accordingly to be immensely distended by some fluid, which, when this bag was opened, was found to be blood, partly liquid and partly coagulated: the quantity was not less than three pints. It was purely blood, without the admixture of any fluid indicating inflammatory action. The external surface of the heart, and internal surface of the pericardium, were examined carefully, but no ruptured vessels, from which the blood might have flowed, were discoverable on either of these surfaces. The heart itself was perfectly sound, the valves were in good condition, and no disease existed in any of the large vessels. The lungs were free from adhesions, and were every where sound. The other viscera were in a sound state. A great deal of care and time were expended in trying to discover the source from which the blood had flowed into the pericardium, but in vain: a slight ecchymosis was observed about the root of the pulmonary artery. Dr. Baillie, in his *Morbid Anatomy*, says, 'Cases have occurred, though very rarely, in which a large quantity of blood has been accumulated in the cavity of the pericardium, but where no rupture could be discovered after the most diligent search, either in the heart itself, or in any of its vessels. This appears very wonderful, and not at all what any person would expect *à priori*. Two conjectures have occurred to me, to explain this phenomenon: 1st, that the blood-vessels on the surface of the heart have lost their compactness of tissue, so that the blood may have escaped by transudation. The other is, that the blood may have been poured out by the extremities of the small vessels, opening on the surface of that part chiefly of the pericardium forming the immediate cover of the heart, from their orifices having been to a very uncommon degree relaxed.'

There is a case related by Dr. Alston, in the 6th volume of the Edinburgh Medical Essays, in which the disease of the chest was of long standing. Three pints of blood, which was partly coagulated and partly mixed with lymph, were found in the pericardium. No ruptured vessel was discovered either on the outer surface of the heart, or the inner surface of the pericardium. Upon pressing the heart, a bloody serum oozed out of a great many orifices on its surface, and principally near its base. No disease was discovered in the interior of the heart or large vessels. Dr. Baillie refers to two cases of extravasation of blood into the cavity of the pericardium, in which the source of the hæmorrhage could not, after the most careful examination, be discovered. In both these, functional disease of the heart had been observed for some time previous to the death of the patient. Vide Medical Observer, vol 10, p. 330. Memoirs of Medical Society, vol. 1, p. 238.

Various opinions have been advanced respecting the sources from which, in the above cases, the blood was derived. One of the suppositions made by Dr. Baillie appears to me to approach the nearest to the truth, which is that the blood had oozed out of the small vessels on the internal surface of the pericardium immediately covering the heart. It is probable, I think, that the oozing, particularly in the case now narrated, arose from the condition of the blood, and the relaxed state of the fibres. It would appear that the disease was general, and that the shivering, faintness, and depression of spirits were not the effects of the flow of blood into the pericardium, but that this last was, like the affections stated, the effect or symptom of the general disease, —that in fact there existed a morbid state of the whole system, similar to that which takes place in purpura, in some kinds of epistaxis, hæmatemesis, and in bleeding from the bowels in typhus fever. The pain in the chest was in the first place, occasioned by the admission of blood into a cavity not accustomed to the stimulus of that fluid. There is no reason to suppose that the action of the heart would be mechanically affected until the quantity of the blood was pretty considerable; for the blood would readily follow the dilatation of the pericardium, occasioned by the elasticity of the lungs, when the chambers of the heart had finished their contractions. No sound was perceived, on carefully examining the chest. Indeed no sound could be excited, as no fluid was poured from one vessel into another. For as the auricles expand as the ventricles contract, the change of place in the constituents of the fluid in the pericardium would be inconsiderable, and made with quietness.

There does not appear to be any symptom in this case that would have warranted the medical attendants in giving an unfavourable prognosis. As a matter of prudence, a less favourable one might have been made, but the same prudence would not permit the expression of a favourable prognosis in any case whatever."

Upon this case we may be permitted to make a remark or two. We have no fault whatever to find with the treatment of the case, nor do we differ from the highly-talented narrator of it as to its pathology—or rather etiology. But we must say that the exploration of the chest was incomplete, and that *percussion,* added to auscultation, would *undoubtedly* have led to a grave prognosis. The state of the pericardium would have caused a dull sound, on *percussion,* over a very unusual space, and would have caused the greatest alarm in the mind of the medical practitioner accustomed to thoracic exploration. The want of any symptoms of enlargement of the heart, too, would have led to the inevitable conclusion that there was fluid of some kind in the pericardium. Thus, then, we see the great value of auscultation and percussion. In the present case, it would have saved the medical attendants from the suspicion (and indeed a justly founded one), that they were unaware of the danger, and consequently of the nature of the disease. Nothing could have saved the life of the patient, had the physician been perfectly acquainted with the true state of the case; but his diagnostic and prognostic skill would not have suffered, had an accurate exploration been made.

We may take this opportunity of saying that, were we to be deprived of one of the two means of exploration, we would far rather give up auscultation than percussion. The latter is far more valuable than the former, though they prove auxiliary to each other.

We are sure that Dr. Carson will excuse these remarks, as they can have no other object than the good of the profession. Dr. C.'s reputation is far above the reach of censure, were we inclined to be censorious, which we are not. His brethren are deeply indebted to him for the candid manner in which he has narrated this interesting case.

---

### TREATMENT OF CHOLERA IN IRELAND.

It appears from a small brochure, by Dr. Wilson, of Belfast, that cholera still lingers in several parts of Ireland, and has re-appeared in places formerly scourged by the epidemic. The object of this pamphlet is to denounce the mercurial treatment of cholera—not because it was less successful than the non-mercurial in its immediate effects; but on account of the injurious or fatal CONSEQUENCES which resulted to those who recovered from the disease. The following extract will illustrate the author's positions and opinions.

"In the hospital (Belfast), the general plan of treatment consisted in bleeding the patient, if young and robust; then administering 10, 15, or 20 grs. calomel, with one or two grains of opium, repeated after various intervals, and washing all down with spirits and water. In the town, bleeding was scarcely ever had recourse to; and, as will be perceived by the quantity used (5 drachms among 1,656 patients), calomel very sparingly. The mortality in the hospital was three times greater than that in the town. It is conceded, that the greater proportional mortality of the hospital over the town, is accounted for by the greater severity of the cases which would be, of course, received there; and I am not disposed, like some others, to lay any of it to the charge of the treatment. The earlier application of remedies, and the generally more favourable aspect of the cases, may be assigned as the reason of the comparative low rate of mortality in the town; and, partial as I am to the town treatment, and, in the same degree, hostile to the hospital, if I examine rigorously the circumstances under which each system was pursued, I can arrive at no other conclusion than that both modes are on a par, so far as the final issue of life or death is concerned; or, in plainer and more intelligible language, that no greater number of cures will be effected by the one than by the other. But, in regard to the CONSEQUENCES of the different treatments to those who survive, I must emphatically denounce the calomel, or hospital treatment, as immeasurably more destructive to the constitution. While we admit, that the greatest proportion of bad cases ought to be charged to the hospital, it is no less true, that many patients, with very mild or premonitory symptoms, found their way there also; for the instructions of the Board of Health to the medical inspectors were, to forward all cases, whether *incipient* or *advanced*, with the least possible delay, to the hospital, and to take charge of those patients at their own homes, who could not be prevailed upon to go there. At the commencement of the epidemic, and through the whole course of it, as was to be expected, many patients, with symptoms generated by terror, and not to be distinguished from the genuine disease, hurried in;—and they, I presume, with all others labouring under the slighter forms of the disease, were subjected to the mercurial plan, which I have so strenuously condemned. From peculiar circumstances, the inhabitants of some districts of the town readily availed themselves of the hospital accommodation; while, in others, none, or very few, of those affected, went in. In one of these latter, an old naval surgeon, who acted as medical inspector, has recorded the names, ages, residences, and *symptoms* of 253 patients, treated by him on the NON-MERCURIAL plan, and the deaths were only 18. I will here give the analysis of this document, which is among the records of the 'Belfast Board of Health.'

110 patients had vomiting, purging, and cramps.
113 patients had vomiting and purging.
18 patients had vomiting & cramps.
6 patients had vomiting.
6 patients had griping, nausea, & slight cramps.

253

From this summary it will appear, that Cholera was met with, and successfully treated, out of doors, on a plan essentially different from that of the hospital; and he would be guilty of great hardihood, who would, after inspecting this list, pronounce that the genuine disease was only to be found in the hospital. Had these 253 patients been treated with calomel, many of them would not now have to congratulate themselves on the integrity of their constitutions, and their freedom from all the deleterious effects of mercurial irritation. The plan pursued with them embraced the use of opium, in a solid form, combined with camphor and Cayenne pepper; of laudanum, in conjunction with sulphuric æther and peppermint water; and of a cordial mixture, the basis of which was whiskey."

Dr. Wilson, in his zeal to banish mercury from the methodus medendi of cholera, has overstepped the boundaries of prudence in his anathemas against this useful, but perhaps often abused drug. He says the Army Medical Board has nearly banished mercury from the treatment of syphilis. Indeed! Dr. Wilson is somewhat behind the march of intellect on this point. The army surgeons are now treating venereal diseases in much the same manner as their *most intelligent* brethren in private practice—namely, by a judicious use of mercury. It is very true, however, that the anti-mercurial mania which prevailed several years ago, and which still lingers among a considerable mass of the profession, is enriching most rapidly a class of sharp-sighted surgeons and physicians, who are widely employed in treating the "CONSEQUENCES"—not of mercury, but of the neglect of of it.

Dr. W. thinks he uses a powerful argument against mercury, when he avers that many invalids come home from India with their constitutions ruined by mercury. We admit this fact, but we meet it by another, which Dr. Wilson has prudently, though, perhaps, not quite candidly, overlooked, viz. that for one constitution ruined in India by mercury, ten are preserved by it from the deleterious influence of the climate. Dr. W. leans on another cogent fact against mercury. "Why is it, at the present moment, that in every college and in every town in the empire, we meet with one or two *veteran* practitioners, who are gradually expelling mercury from the list of their medicinal agents?" We can tell Dr. Wilson why and how this is. It is because in every college and town in the empire, there may be even more than one or two *veteran* practitioners who are passing into the state of second childhood, and parting with what little intellect it pleased God to give them originally.

---

### Composition of the Pontine Marshes.

The following is the concise account of these celebrated seats of malaria by Dr. Weatherhead, who walked across them rather than use the legs of the quadrupeds who carry our nobility and gentry with such speed across these pestilent quagmires.

"The volume of water which escapes from under the lime-stone mountains of the Apennines is truly astonishing. The principal drains run on each side of the road, and more resemble wide canals than drains in the ordinary acceptation of the word. They are so well levelled that the stream of water cannot stagnate, but runs freely away. With the object of ascertaining the nature of the soil of these celebrated marshes, I made excursions to the right and left of the road, where the water allowed me; and the result of my observations surprised me a good deal. The soil in most places is exceedingly superficial, often not deeper than two or three inches; and below this there is a foundation of solid stone. This last is a calcareous deposition from the waters flowing from under the mountains, and is precisely similar to the travertine found and formed in the neighbourhood of Tivoli. This sediment encases, and, in time, petrifies, the reeds and other tubular vegetables that grow in the soil, thus forming congeries of interrupted conduits for the lodgement of water. It is to this peculiarity of formation that the miasmata of the Pontine Marshes, in great part, owe their origin; but while there is no denying its pernicious influence to a certain extent, the degree of alarm this excites appears to me one of those common errors perpetuated by idle repetition, unconfirmed by personal investigation, and unreasonably exaggerated by the fears of the pusillanimous. In my way I passed above forty labourers at work, widening one of the drains; and, as far as I could judge by appearances, they all seemed robust and healthy, working with vigour under a scorching sun, and half up to their knees in water. Habit, it is true, is Nature's lieutenant, and we see elsewhere indigenæ thrive in a climate which is almost certainly fatal to a stranger not inured to it. Late in the evening I arrived at Terracina, where a comfortable bed and supper wore off the fatigue of the preceding day's march."

To Dr. Weatherhead's account of the composition of the Pontine Fens we do not demur; but we cannot say that the aspect of the inhabitants made the same impression on us that it did on our pedestrian traveller. The workmen in these morasses are mostly convicts, and those which our author saw may have been a fresh batch from the Papal prisons or the mountains. But we venture to say, that no people would long retain the semblance of health in such localities.

---

Ligature of the Carotid Artery.

Our readers will remember some notice which we took of Mr. Preston's case—ligature of the carotid artery for epilepsy. In the last volume of the Calcutta Transactions, we find Mr. Preston renewing the subject; and he there informs us that the epileptic patient on whom he operated in February, 1831, has not had any return of his epilepsy, and is in perfect health. Another patient also, on whom the operation was performed, has regained, in a great degree, the use of the paralytic arm, and is quite free from pain in it. In the present paper Mr. P. takes occasion to observe that the operation in question is applicable to more affections than epilepsy. We shall now take a glance at some cases detailed in this volume.

Case 1. *Headache and partial Paralysis.* This was a robust man, a native trumpeter, aged 24 years, admitted into Garrison hospital, at Cuddalore, 10th of August, 1831. He had been in hospital several times before, once for intermittent fever, and afterwards for headache.

"The symptoms under which he at present suffers are as follow: constant pain in the head, particularly the fore part; when he speaks his mouth and face are drawn to the right side; although his muscles are large, he is extremely feeble, and unable to walk without support; there is an appearance of idiotcy in the expression of his countenance and manner, but his judgment is not impaired; his stools frequently pass away involuntarily; there is partial loss of motion in the left arm and leg; complete loss of vision of the right eye, without any apparent change of structure; this he says was produced by inflammation consequent upon a blow; vision of the left eye greatly impaired; wanders very much at night; evacuations natural.

A very slight and temporary relief was obtained by the exhibition of iodine, by a seaton to the neck, and a blister to the head. His present symptoms are precisely those detailed above, and appear to me attributable to increased arterial action within the cranium; with a view of diminishing which I have determined after much consideration, upon tying the right common carotid artery."

The right carotid artery was accordingly tied on the 2d of September, and on the seventh he was free from headache—was much stronger in his limbs, and had lost the appearance of imbecility. He continued however to shew some disposition to delirium at night. He had not the proper control over his facial muscles, and could not command the expression of the face. On the 27th he walked five miles, and was much fatigued. This was followed by some fever. His vision was impaired.

"On the 3d of October, his vision was not improved, although the head was quite free: he was ordered calomel and opium three times a day. On the 6th there was slight salivation, but no improvement of vision. As the affection of the sight was attributed to some return of the state of the brain for which the first operation was performed; a ligature was applied to the left common carotid artery on the 10th of October. He felt very weak after the operation, although very little blood was lost. He was discharged on the 11th of November: vision continued imperfect; but in every other respect he had entirely recovered his health. He was subsequently discharged, on account of the defect of vision."

The above was what we would call bold practice.

*Case* 2. A man aged 51 years, was admitted on the 22d August, 1831. He was a short stout man with very short neck, and its muscles much contracted, so as to draw the head down upon the chest. The eyes were full, prominent, and had a strained appearance. This man had been subject to epileptic fits (coming on six or seven times in the month) for six years past.

"His present symptoms are, complete loss of power of the right half of the body, with very great impediment to utterance; the muscles of the face and tongue are drawn to the left side when put in action. This attack of hemiplegia occurred twenty days ago, immediately previous to which he had been drinking hard. The feet are cold; great irritability of temper is evident; the right thumb is bent upon itself, and kept down upon the palm of the hand; if straighted, it becomes bent again immediately, in the same manner: the thumb has been in this state for the last seven weeks, having assumed this position immediately after an epileptic fit. The pulse and skin are natural; there is now some pain in the head, and he has for a long time suffered from head-ache. I do not conceive that any benefit is to be expected from the usual modes of treatment in this case, neither does the continuance of this state appear to me free from danger. Intellectual functions not affected."

A ligature was placed on the right carotid artery, 23d of August. On the 24th he could move both arm and leg and straighten his thumb, without assistance from the other hand. Less head-ache than before the operation, and speaks better. By the 3d of September he was able to move about with the assistance of two sticks. On the fourth he had three epileptic fits, and was rather worse the next day. After some vicissitudes in the course of his complaint, he was discharged greatly improved on the 26th September, being able to walk about with a stick, and free from fits or head-ache. On the 14th of November, however, he was readmitted, having previously suffered much from epileptic fits. There had also been much head-ache—inability to walk—constant throbbing in the head—voice much affected—the fits much increased in violence at full and change of moon. The left carotid artery was therefore tied. He was previously bled and leeched with some advantage. On the 16th November, two days after the operation, we find him complaining much of the head, and the power of the right arm almost entirely gone.—Nov. 18th. Much better—has recovered the use of the right arm. On the 23d, a seton was inserted in the neck. Afterwards nux vomica, a grain three times a day, was given, and whether from this, from the seton, or from both these with the operation, he appeared to be much better. On the 8th December he was discharged, though not cured. On the 25th January, 1832, we are informed that the unfortunate man still suffers from pain and throbbing in the head—fits—inability to speak—and much difficulty in walking. Our author thinks that his life has been prolonged by the operations, and that many of the symptoms have been mitigated. There can be no doubt of serious and insuperable organic disease of the brain, which will, ere long, terminate his existence.

P. S. A short report is appended, dated 17th January, 1833, in which we find that the patient had much recruited, but was seized with paralysis agitans. From this, too, he recovered considerably—and "is now able to walk about with one stick, with ease, and speaks with very little impediment."

These cases are certainly curious both in a physiological and pathological point of view, and deserve the attention of our brethren.

---

### THE ABSENTEE; OR, BLESSINGS OF ITALY.

The following portrait is drawn by the Author of the "RECESS," in the pump-room at Cheltenham. It is said to be imaginary; but we believe that it is drawn from real life.

" A tall gentleman, with two ladies, (apparently his wife and daughter,) advanced to the pump, and each drank off a goblet of the medicinal waters. There was a peculiar unhealthiness in the aspect of these three individuals, which attracted my notice. It differed from the half-jaundiced sallowness of the Anglo-Indian invalid—and also from the pallid and faded complexion of the fashionable and dissipated SEASONERS of the metropolis. It had a sickliness, *sui generis*, and beyond my power of analysis. As soon as they began to converse with each other, I recognized the accent of the Emerald

Isle—and this increased my wonder. I applied to my aerial CICERONE for information. 'That gentleman,' said he, 'is a great landlord and squire in the county of ——, Ireland. His numerous tenantry are ignorant, and therefore semi-savage—disagreeable to the eyes of the fashionable family; and therefore, perhaps, somewhat dangerous. Under these circumstances, it was opportunely discovered that the health of a daughter was delicate, and that the climate of Ireland was damp—that the skies of Italy were bright—the society there recherché—and expenses very little more than in Castle Rackrent. The conduct to be pursued admitted of no question. The bailiff was ordered to collect the rents, and the courier was ordered to prepare for the journey. Paris was visited—the Simplon was scaled—and Florence, Rome, and Naples were explored.—Years passed away on the classic soil, and yet curiosity was not sated, nor pleasure exhausted. But, on a fine summer's evening, while the family were sitting on the heights of Albano, inhaling the balmy zephyrs, and enjoying the superb panorama of the Campagna, with its scattered ruins and surrounding Apennines, one of the young ladies inspired the deadly poison that so often floats on the fragrance of Italian gales! The tide of happiness, like that of fortune, has its affluxes and effluxes. The current of affliction now took its turn. The hapless and innocent victim of an Italian climate, (to speak of nothing else,) fevered, faded, and ultimately sunk beneath the pestiferous influence of the syren soil! Her spirit fled to heaven—her mortal remains lie on the banks of the Tyber, near the pyramid of Caius Cestius.

'The long inhaled poison of Italian malaria—the heart-rending scenes attendant on the protracted sufferings of the beautiful girl—and feelings, which are best known to the unhappy survivors, have produced the frightful ravages in the minds and bodies of the party before you, which have rivetted your attention. The waters of Lethe may, but those of Cheltenham never can, wash out the mental and corporeal sufferings of that wretched TRIO.'"

### Curious Geological Phenomena.

Dr. W. Beattie is conducting the letter-press of a very interesting, able, and cheap work, "SWITZERLAND ILLUSTRATED," from the second number of which we extract the following geological particulars. Near Geneva stand two mountains, the great and the little Saleve, presenting their steep escarpments of lime-stone to the valley of the Rhone, while, on the South-side, they slope down to the valley of the Arve. On this (South) side, may be seen, not the remains of an ancient city or temple, but the magnificent ruins of mighty mountains, and the monuments of an overwhelming catastrophe, which transported these ruins into their present situation. The snow-clad mountains from which they were torn rise majestically to view, at the distance of 50 miles! On the little SALEVE, 1400 feet above the valley, are scattered numerous blocks of granite, of vast size, not at all water-worn, but almost as fresh as if recently rent from their parent mountain. They are identical with the granite of Mont Blanc, while the surrounding mountains, and the Saleve itself, on which they lie, are *calcareous!* On the great Saleve, and 2500 feet high, is a huge block of this granite. These blocks are not broken or shattered, as they would have been, had they been hurled with violence from the Alps; neither do the lime-stone strata, on which they rest, present any appearance of fracture or indentation by their fall. The blocks lie quietly on the surface. Dr. Beattie inclines to the explanation attempted by Bakewell,—namely, that these blocks were transported by the floating glaciers of the Alps, and not by any sudden rush of waters, as Saussure imagined.

We strongly recommend this work. Each number contains four beautifully executed plates, besides the letter-press, price only *two shillings*.

---

### The Sirocco.

Dr. Weatherhead has broached a new theory of the sirocco. In the first place, however, he avers that nothing is more indefinite than the word SIROCCO, "since every breath of air offensive or oppressive to a travelling Smellfungus's feelings must assuredly be a sirocco." Against the idea of this debilitating and suffocating wind being caused by its passage over the Afric sands, our author opposes the fact that Agrigentum and the whole south side of Sicily, which are directly opposite to the point whence the sirocco is supposed to come, "is not noticed by any writer as being particularly exposed to its stifling influence." Naples, Messina, and Palermo are, he says, the places principally annoyed by the sirocco. "Again, no part of the coast of Greece has ever been reputed as liable to the effects of the sirocco." Finally, our author denies that the sirocco is a wind at all—it is a calm! Now with respect to Greece, we find Homer talking of—

——————— Auster's sultry breath,
Pregnant with plagues, and shedding seeds of death.

While Horace, more than once alludes to the "plumbeus auster." Mr. Matthews pretty significantly answers the objection of Dr. Weatherhead as to the sirocco being a wind at all—in the following passage.

"The sirocco wind, which has been blowing for six days, continues with the same *violence.* The effects of this south-east *blast,* fraught with all the plagues of the deserts of Africa, are immediately felt in that leaden oppressive dejection of spirits which is the most intolerable of diseases."

Dr. Johnson, too, bears testimony to the truth of Mr. Matthews's description of the sirocco *wind.*

"Yesterday the sirocco—Auster's sultry breath—steamed over Naples, depressing the animal spirits and the vital energies to the lowest ebb. It is impossible to convey an adequate idea, in words, of the sedative effects of this wind on mind as well as body. I tried to respire in freedom on the roof of the Vittoria—on the Chiaja—the Mole—the Cheatomone; but found no relief from the nervous depression and muscular languor induced by this mephitic composition of rarefied air and aqueous exhalation. From lassitude of body and dejection of mind there was no escape, while this accursed *blast* prevailed."—*Change of Air,* 3d *Edition.*

We do not deny, however, that the depressing effects above-mentioned may be felt during a calm; but we believe that such calm must be posterior to a southernly wind—or that a stream of air from the South actually prevails at some height, though the atmosphere appears calm on the surface of the Earth. We shall allow Dr. Weatherhead, however, to state his own theory of the sirocco.

"From the fuming mouths and crevices of Vesuvius, and the pseudo-volcanic vicinity of the Pisciarelli, Solfatara, and Baiæ *(vaporiferæ Baiæ,)* from Stromboli and Etna, there is constantly issuing mephitic vapours and gases, which, from their heated and rarefied state, naturally ascend, and, mixing with the purer circumambient air, get diluted and dispersed by every casual wind. But let us suppose not an unusual occurrence to take place, namely, that this mephitic atmosphere shall suffer a sudden diminution of its elasticity through a change of temperature taking place high up in the air, while the aqueous vapour it holds dissolved becomes in consequence more condensed; and that at the same time there shall be no wind to disperse the gaseous exhalations as they continue to arise from below; the natural effect must be, for these dense vapours to descend, and for those which are being evolved to fall again, as soon as they have cooled down to an equilibrium of temperature with the surrounding atmosphere. The necessary consequence of all which must be, for this concentrated mass of mephitic vapour to lodge, by reason of its greater specific gravity, on the surface of the earth, and thus envelope within its range and influence every being that breathes."

If a *calm* be essential to the Doctor's theory, we can only say that a breeze of wind is *very generally* the accompaniment to a sirocco, even of the worst kind—and if this be a fact, the hypothesis of our worthy and ingenious traveller is in some danger.

### Ulcers of the Leg.

From a little volume of considerable practical utility, entitled "Observations on the Ulcerative Process and its Treatment, particularly when affecting the Leg," by Mr. Eccles, we make the following extract, as a sample of the matter and manner of this unostentatious publication.

"The cases in which the morbid condition of the condensed cellular tissue forming the cup of the ulcer appears to be the cause of continuance of the disease, are distinguished by the induration of the edges, the scantiness of the secreted manner, and the insensibility of the sore. There can be little doubt that bandaging and various stimuli have some effect on the cellular structure in question, as well as on the sanguineous and secernent vessels; but there are two agents which I find of particular efficacy in removing the more obstinate peculiarities of such cases. These are mercury and iodine. The former is well known to possess the power of lessening induration when given internally, and some have thought that the same efficacy is attached to its local application. After much experience, I am, however, brought to the conclusion, that mercurial dressings are void of any specific utility analagous to the constitutional action of the remedy; that they stimulate in peculiar modes, but perform no important part in effecting the removal of indurated cellular tissue. The local effect of mercury is extremely great in changing the character of sores in general, and in the form I am describing, it is very salutary. We shall find, however, that if its action be not immediate, no good purpose will be answered by its continued employment.

Iodine, internally administered or externally applied, and even in those forms which have lately been described as inefficacious, I have always found of great service in removing this species of induration. It often requires to be used for a tedious length of time, to be remitted should it produce much constitutional derangement, and again employed when the state of the general health will permit. I have not met with a case in which iodine has failed to remove the induration; but if such were to occur, it would perhaps be advisable to remove the indurated structure with the knife. This has been done, and certainly with perfect success, the new wound healing rapidly. But so decisive a practice would only be justifiable where every other method had repeatedly failed."

There are many judicious observations in this book on the ulcerative process, as it affects the different animal structures, which deserve the attention of the young surgeon.

---

### Dr. Weatherhead on Malaria.

In an amusing and instructive pedestrian tour through Italy, by Dr. Weatherhead, we find the following conjectures (for they can hardly be considered as proofs) relative to the origin of this mysterious agent in the eternal city.

"Walking on the Monte Pincio one day, I perceived thin and variously composed strata of volcanic dust, developed by the partial cutting away of the hill for the path which ranges on its height; and on examining it in different places, I found it to be entirely formed of a mound of the same volcanic material. It is of a bluish colour, speckled with white spots perfectly calcined, and possesses a strong attraction for humidity. Some that I got several months ago is even now more damp than when taken from the hill, though repeatedly dried by the sun as carried about in my knapsack. This property of the soil of Rome is, in my opinion, the chief source of the malaria, so fatal in its effects here at certain seasons of the year. Its line of distribution marks the limit of its operation, and this circumstance will explain how one side of a street should be notoriously unhealthy, and the other free of any noxious influence. The most heedless observer must frequently have witnessed how speedily the roads in the neighbourhood of Rome dry after even great torrents of rain. He mistakes much if he thinks this proceeds from evaporation; for the heat of the sun, even in the hottest summer months, could dissipate but little in so short a space of time: it is absorbed by the thirsty nature of the soil; and he may convince himself of the fact, by remarking how permanently moist this is all the year round a few inches under the surface. Heat and moisture, we all know, vivify and disengage the fomites of disease; no wonder, then, that these, acting on the débris of animal and vegetable matter in a state of decomposition, buried for ages, and daily gaining fresh accumulations, should generate pestilential effluvia, and by contaminating the atmosphere of Rome during summer, produce fevers of so fatal a type.

This pernicious condition of the soil is not confined to Rome (six out of seven of the hills on which it stands I ascertained to be volcanic), but extends as far as the deliquescent earth (its peculiar matrix) itself does; and hence the unhealthiness of the whole of the Campagna. Circumstances certainly modify its degree of intensity; but I think facts will bear me out in circumscribing the sphere of the operation of malaria to the demarcation made by the line of its extent. The Pontine Marshes, again, owe any peculiar unhealthiness they possess to another kind of formation, of which I shall speak hereafter."

Dr. Weatherhead's theory does not account for the varying position of the malarious portions of Rome. It is well known that the unhealthy topography changes, and that malaria has, for many years past, been progressing from the eastward to the westward. It cannot be supposed that the soil itself has changed—and we must therefore come to the conclusion that some subterranean agent is at work which causes the emanation of mephitic miasmata from the surface of the earth, while the primary cause is far from our ken.

We cordially agree with Dr. W. however, in the following sentiments.

"The air of Rome is heavy and unwholesome, especially for invalids requiring a strict regimen and great care; and perhaps it would be advisable, on more accounts than one, to have regard to the ordonnances which the stranger will read in the church of Minerva.

With regard to the fitness of the climate as a residence for the pulmonary invalid, I cannot agree in those unqualified commendations which some have bestowed upon it. The air, as I have said, is heavy and moist, and certainly there are some whose lungs such a temperament of atmosphere may suit; but this I think is certain, that if it prove not beneficial, the trial cannot be made with impunity; and no physician, if honest in his opinion, can say, *à priori*, whether it will prove so or not. In spring, again, and even in summer, a cold wind blows at times from the Apennines, which suddenly chills the air. This is an observation of Pliny's. My conviction is, that many a consumptive patient, who might have leisurely walked to the grave elsewhere, gallops to his goal at Rome: his languor increases under the depressing influence of so moist and relaxing an atmosphere; his nocturnal perspirations become more profuse and colliquative, his expectoration more exhaustingly copious; a quickened circulation fans the inflammatory combustion, and a keener hectic feeds on the vital principle until it is consumed, when death, closing the scene, bears away the last sigh, fraught with regret for having ever left home."

---

### TEMPERANCE SOCIETIES.

In the long black catalogue of causes which occasion sickness, death, and crime among mankind, there are few items which appear more conspicuously operative than intemperance—especially intemperance in ardent spirits. It is difficult to say which is most injured by this—the mind or the body. Certain it is, that both the morale and the physique suffer severely from this cause, the amount of moral ill being, at least, equal to the amount of bodily disease. Widespread as the evils of intemperance are in this country, it is acknowledged, both by American and

European travellers, that the diffusion of it is greater beyond the Atlantic than in the Olden Land. Many causes may have conspired to produce this misfortune—or rather this misconduct. The cheapness of the inebriating material must be one. Example spreads the pestilence, and it is not much to be wondered at that the myriads of emigrants from Ireland, Scotland, and other parts of the world into the Land of Liberty and Plenty, should have given an immense impetus to the habit of intoxication. But no doubt a great variety of causes have combined to produce the evil in question, and we are happy to learn that a corrective check is springing up among our transatlantic brethren, which bids fair to grapple successfully with the enemy, by attacking him in his very citadel, and strangling him in birth. These observations will be elucidated by the following letter from Dr. John S. Butler, of Worcester, Massachusetts, accompanying a number of documents, which we are unable at this moment to analyze and collate. We have seen enough however, to certify that the letter of our brother physician gives a fair resumé of their principal results, and that in clear and forcible language. The intelligence will be particularly gratifying to every friend of religion, morality, and social order in this country.

Worcester, Massachusetts, N.S.A.
April, 2, 1834.

Dr. James Johnson.

Sir—The devotion of the pages of the Medico-Chirurgical Review to the promotion of science—its high-toned liberality—the fearlessness with which it assails ancient abuses—the power with which it exposes popular fallacies—its wide spread and happy influences, and the energy and zeal with which it brings those influences to bear upon the public good, with the kindliness and courtesy which it manifests towards the institutions, &c. of America, have induced me to intrude upon your attention the accompanying documents, and to solicit for them a brief consideration.

They relate to the history, present condition, and prospects of the Temperance *Reformation* (as the popular movement against the use of ardent spirits has been aptly termed) a subject which has excited an intense interest in the United States, and one which must exert an important influence upon the practice of medicine and surgery in all parts of the world.

The public mind in England has also been called to it in some degree; but aware that much of error and misrepresentation has ever been cast around this enterprize both *here* and elsewhere, and fearing that the friends of the cause in Great Britain may be induced to trust to the power of legislation rather than to the mightier energies of *enlightened* public sentiment, I present these documents to you, Sir, in order that these principles may be fully and fairly presented to your mind, with the expression of my earnest solicitude that you may be induced to appear the same able and eloquent advocate of *social* as of *medical reform*. Referring you to these documents for a full exposition of this subject, permit me briefly to state a few prominent and leading facts.

It was about ten years ago that the public attention in this country was first aroused to the alarming extent of the vice of intemperance. Careful investigations, made at different times and at different places, by gentlemen of patriotism and philanthropy, *proved*, by the most conclusive and undeniable evidence that "two-thirds of our pauperism, nine-tenths of our crime, two-thirds of our insanity, and an annual expense of from *one hundred to one hundred and twenty millions* of dollars were to be traced directly or indirectly to the moderate or immoderate use of ardent spirit; that 300,000 drunkards, 75,000 criminals, 200,000 paupers, 6000 lunatics, were its victims—whilst during every year, at least 30,000 sunk into the drunkard's grave. It was found that the ravages of this vice had extended not only to the sanctuaries of religion, our legislative halls and judicial courts, but that the freedom and purity of the elective franchise was endangered."

Voluntary associations pledged to total abstinence, the diffusion of intelligence in every way by the publication (and in many instances, gratuitous distribution) of essays, newspapers and tracts,—by public discussion in assemblies and conversations—by addresses from clergymen, &c. and by agents traversing the country in every direction, have been the means with which we have endeavoured to drive this fearful pestilence from our borders. The press of the New York State Temperance Society alone, has issued during the past year 4,438,500 copies of Temperance publications; equal to *eighty millions* of duodecimo pages!

The Massachusetts State Temperance Society, formed in 1826, was the first society, (of any importance) organised in this country. According to the 6th Report of the American Temperance Society, published in *May*, 1833, since 1826, 6,000 Temperance Societies have been formed, 2,000 distilleries have been relinquished, 5,000 merchants have abandoned the traffic, 5,000 drunkards have become sober men (many of them highly respectable and useful members of society), 700 vessels are navigated without the use of ardent spirits, and upon all such vessels the Insurance Offices of Boston deduct 5 per cent. from the premium of insurance, and more than one million persons have signed the Temperance pledge!

The use of spirits is forbidden in the army and nearly abandoned in the navy. Ten months have passed since the above meeting, and during this time the reformation has advanced with unparalleled rapidity; it is now estimated that there are *more* than 1,500,000 signers to the pledge; unquestionably the next Annual Report will shew an increase of the above statistics of *at least* 33 per cent. The February No. of the Temperance Magazine will contain the signatures of 1,600, nearly 2,000 in all, American and English, of the most respectable physicians in the N.S. to the statement that "men in health are never benefited by the use of ardent spirits," and that "the use of them is a frequent cause of disease and death."—[*Vide* p. 8 of 6th Report A.T.S. and No. 1. Vol. II. T. Mag.]

I will mention but one fact more and that in regard to the influence which this reformation must have upon the practice of medicine, &c. The population of Albany is 26,000 of whom 5,000 are members of the Temperance Society—number of deaths by cholera in 1832, 336 reported—of whom only two were members of the Temperance Society! The documents to which I would refer you for evidence of the accuracy of the statements and for more full and explicit accounts, are the 4th, 5th, and 6th Reports of the American Temperance Society, the 1st vol. of the Temperance Quarterly Magazine, the 1st No. of the 2d vol., and some copies of the Recorder, Intelligence Almanac, &c. Several important documents will come out in the course of the Summer: it will give me great pleasure to forward them to you from time to time, if it would be agreeable to you to receive them.

In this country, at least, the profession knows no higher authority than the Medico-Chirurgical Review. Its influence and its circulation are co-extensive with the science of medicine, in all parts of the world. Its influence, therefore, is as wide spread as it is salutary. It appears to me that the subject of the Temperance reform comes strictly within its legitimate sphere; and were it to come out and expose the consequences of intemperance—*of the use of ardent spirits as a drink*, as ably as it has the ignorance of quackery, the folly of cholera-phobia, and the corruption and bigotry of corporate monopoly, it would do a more blessed deed for *our* "father land," for the *world!* than it is in the power of any other individual in the British Empire to accomplish.

Pardon me, Sir, if I have appeared to have forgotten that I am addressing a stranger—the Editor of the Medico-Chirurgical Review hardly appears to *me* in that character! I have so often turned to his eagerly welcome pages for delight and instruction; have been so often guided aright by his advice, in those hours of anxiety and doubt which so often hang over the *young* practitioner, that I must confess some of the feeling as towards an elder and more experienced practitioner, upon whose sympathy and kindness I have been accustomed to rely.

It may not be in my power to send the last number of the Magazine, (No. 1, of Vol. 2d) if it does not come to hand in time for the packet. I will send it by the next. If you should desire to obtain more information upon this subject, or upon *any subject* in relation to this country, I shall be proud to have you command my services. Sensible that your time is too valuable to be wasted, I have ventured to mark those portions of these documents as will probably be most interesting to you. If they are safely received, it will be gratifying to me to receive a brief acknowledgment of the fact. Please address to Dr. John S. Butler, Worcester, Massachusetts, N.S.A.

If further apology is due for the boldness with which I address you, Sir, let it be found in the ardour of *youth*, and its necessary sympathy with the generous sentiments which pervade your writings.

I have also sent you two Reports of the Trustees of the Massachusetts State Lunatic Hospital—a hospital for the *pauper insane!* Similar institutions are about being erected by several other States—this is the only one of the kind in the N.S., and I trust that the noble example which the humanity of Massachusetts has placed before the public, will ere long be imitated by every State in the Union. You will perceive that here the pauper has the first claim, and there are so many of that class, that nearly all of the private patients have been dismissed for want of room.

I have the honor to be, Sir,
Your obedient Servant,
John S. Butler.

We beg to return Dr. Butler our best thanks for his interesting communication, and sincerely hope it will have all the beneficial influence in this country which his own philanthropic mind can hope or desire.—Editors.

---

### Provincial Hospitals.

We willingly give insertion to the following memorial from the Bristol Infirmary, because it bears upon the medical polity of the provincial hospitals and schools generally.

"*To the Chairman and Committee on Medical Education, &c. &c.*

Gentlemen,

1. In addition to the answers to the questions contained in your Circular, we beg leave to subjoin the following statement, to which we respectfully call your attention, as connected with the subject which must form no inconsiderable portion of your inquiries, namely, *Medical Education.*

2. In the year 1735, Bristol projected, and in 1736 carried into effect, the scheme of a Hospital, supported entirely by voluntary contributions. Its foundation is believed to be nearly coeval with, if not to have preceded, that of the Hospitals in London, and of similar Institutions in Scotland and Ireland; royal, endowed, and chartered foundations only excepted. This Institution now contains from 200 to 220 In-patients—Out-patients, from 5,000 to 6,000. Average number of casualties, about 1,200 annually.

3. The whole range of Building is uniform and extensive, having been re-erected within these few years; furnished with Baths, Warm Air, and other modern conveniences; and has attached to it a spacious garden, as a promenade for convalescents; in a word no expense has been spared which could contribute to the comfort and recovery of the inmates.

4. This Institution is also enriched with a valuable and daily-increasing Museum, a Library, a Lecturing Theatre, and excellent accommodation for Examinations and Dissections; in the latter of which our students are almost daily engaged, under the able superintendence of the House-Surgeon.

5. The ordinary disbursements are annually from £5,000 to £6,000; and there has been lately expended, in providing accommodation for the Out-patients, about £3,000 in a supplementary building.

6. It may not be amiss here to recount the advantages which the city of Bristol possesses for a great Medical

Establishment. With a population equal to that of most of the capitals of Europe—with a large and well regulated Hospital, and also a Medical School—the student here may learn not only the principles and practice of Medicine and Surgery, but also witness, on a great scale, the nature of most of the diseases and accidents which afflict mankind.

7. With the above statement before you, gentlemen, it will, perhaps, be a matter of surprise, that there could have existed any doubt of the propriety of conceding to us the ability to qualify any students for examination; yet the fact is, that it is only since the *25th of November*, 1831, that we have been '*recognised*' by the College of Surgeons; and even then partially, as our dressing apprentices of *five* years' standing are at present under a peremptory *obligation* of walking, as it is termed, some one or other of the London Hospitals, for a period of six months. On this subject it is remarkable, that the regulation of the College should only require a *walking* attendance of twelve months in a London Hospital, from young men who never before may have been within the walls of any Hospital, while our apprentices have not only winessed the practice of the Bristol Infirmary, but have been constantly employed as *dressing* pupils, and in the charge of casualties, for a period of *five* years.

8. This representation, we presume, entitles the Bristol Infirmary to be justly considered a hospital of the first class. Even in the metropolis there are not above four or five that exceed it, either in size or importance, as a school for scientific and practical surgery; and none surpass it as to the regulations established by its governors for the education of the students; it may be asserted that no hospital in the kingdom affords greater opportunities for the acquirement of medical and surgical knowledge. On this subject the surgeons are desirous of the fullest investigation; and, as some proof, they beg to refer to every one of their students who have attended in London, who will confirm the fact from their own experience and observation. Besides, it is well known, that students, who have received their medical education at this Infirmary, have acquitted themselves with marked ability as candidates for their diplomas, when under examination at the College of Surgeons and Apothecaries' Hall.

9. For these and many other reasons, the surgeons of the Bristol Infirmary have ever considered the following regulation of the Council of the Royal College of surgeons as oppressive and unjust; but they are willing to believe, founded on an erroneous view of the opportunities the Bristol Infirmary affords of cultivating medical science. Pupils and apprentices, when candidates for the diploma, must bring proof—

10. '*Of having attended, during twelve months, the surgical practice of a recognized Hospital in London, Dublin, Edinburgh, Glasgow, or Aberdeen; or for Six Months in any one of such Hospitals, and Twelve Months in any recognized Provincial Hospital.*'

11. The surgeons of the Bristol Infirmary are of opinion that the above regulation of the Royal College of Surgeons,—which requires an attendance of Six Months, as a Walking Pupil, at one of the Hospitals in London, in addition to an attendance during one or more years at a recognized Provincial Hospital, as a necessary condition for obtaining the Diploma of the College,—is highly objectionable; and for the following reasons:

1st. Because it is unnecessary. A student may spend the prescribed time quite as profitably in attendance at a recognized provincial Hospital, as upon those in London. In fact, with the exception of four or five of the metropolitan Hospitals, the recognized provincial Hospitals are upon a larger scale than those of the metropolis.

2nd. Because it is injurious to the morals of students. It is a notorious fact that students, after a residence of only a few months in the Capital, frequently fall into practices of dissipation and immorality foreign to their previous habits, and ruinous to their future usefulness, respectability, and happiness.

3rd. Because it has a tendency to render provincial education less efficient than it would otherwise be. It has often been observed that students neglect to make a proper use of the advantages afforded to them in their attendance at a recognized Provincial Hospital, from a notion that the full prosecution of their studies may be delayed until their residence in London, where, they imagine, the opportunities of acquiring professional knowledge will be more worthy of their attention; and, in consequence, they crowd into that brief period a far greater number of pursuits than their time, or their previously-formed habits and inclinations will enable them to follow with any prospect of advantage.

4th. Because it occasions a very considerable increase in the pecuniary expenditure of the student, without advantages in any degree correspondent to the extent of the outlay.

Lastly. Because it implies an invidious distinction between the surgeons of the recognized Provincial Hospitals and those of the Metropolitan Institutions, a distinction which cannot be supported by a fair comparative estimate of their respective attainments and capabilities.

In addition we beg to draw your attention to the circumstance that, not only has the time prescribed for Professional Study been of late considerably lengthened, with a corresponding augmentation of expense, but the number of branches of science which must be attended to has also been greatly increased.

Gentlemen, we will take up your time no longer than to submit to you that we require redress; and to repeat that the Bristol Infirmary has a fair claim, in every point of view, for admittance into the first class of Hospitals in the United Kingdom.

We have the honour to be,
Gentlemen,
Your most obedient Servants,
Richard Smith,
William Hetling,
Richard Lowe,
Henry Daniel,
Nathaniel Smith,
*The Surgeons of the Bristol Infirmary.*

Bristol Infirmary Consultation-Room,
April 22nd, 1834.

To Henry Warburton, Esq. M. P. &c. Chairman of the Committee on Medical Education, House of Commons, London."

The injustice of putting a five years' *dresser* at the Bristol Infirmary nearly on a par with those pupils who have never been in an hospital at all, is indeed striking, and will, no doubt, be remedied in the new order of things. The question whether any hospital attendance in London, at all, be necessary or desirable, is not so easily settled. We believe that not one of the medical officers of provincial hospitals would fail to send their sons (if educating for the profession) for ten or twelve months, to a metropolitan hospital, previously to settling in practice. But this does not prove that a London hospital attendance ought to form a sine qua non for passing the College of Surgeons. If the curricula of education be rendered uniform throughout these Islands, and the examinations searching, we would merely suggest a longer attendance on a provincial than on a metropolitan hospital. It ought to be remembered that, in London, the pupils of one hospital have the privilege of seeing the operations in all—a privilege which certainly is not entirely valueless. Many operations may be seen in the course of a year in the London hospitals, which never occur in a provincial establishment—and the very circumstance of seeing the various surgeons of the metropolis operate, is not without its benefit to pupils from the provincial schools.

---

### Dr. Turnbull and Veratria.

In our last No. we expressed a hope that the experience of others might confirm that of the author, promising at the same time that we should communicate the results of our own trials. It is unnecessary to assure our readers that on this, as on every other subject of professional inquiry, our minds have not been biassed either by prejudice or partiality. Truth and justice are ever the only guides of our conduct.

In two cases of facial neuralgia affecting the infra-orbital nerves, and which had continued, with varying severity, for three and seven years respectively, the use of the strong veratria ointment has been followed with speedy and decided relief; the paroxysms being rendered not only of much shorter duration, but also less agonising and of less frequent recurrence. One of the patients, a carpenter, about 60 years of age, had been for nearly two years so afflicted, that he was quite incapable of following his work during almost the whole of that time; every alteration in the state of the weather, however trifling, inducing a fit of pain. After a week's employment of the ointment his condition was greatly improved, and along with the comparative ease which he enjoyed during the day, he began to sleep quietly at night; a comfort of which he had been nearly quite deprived.

The second case, occurring in a countryman, of between 60 and 70 years of age, was as satisfactory as the preceding. Both patients are indeed still subject to returns of the enemy's attack; but so confident are they of having him under their power (by rubbing the affected parts with the ointment until its full effects are produced) that they always carry a box of it in their pockets wherever they go.

We have seen a third case of painful affection of some of the twigs of the infra-orbital nerve, in a middle-aged lady. It had resisted a great variety of treatment; but has now yielded almost entirely to the use of the veratria. From the circumstance however of this patient being of an extremely nervous and delicate constitution, as well as from the other features of the case, we were inclined to regard the facial pain, rather as one symptom of hysterical disease, than as a specimen of genuine or idiopathic neuralgia. Nevertheless we must confess that it had tortured her for a length of time, and that the relief which she now enjoys is truly great.

The fourth case was one of rheumatic neuralgia, affecting the lower extremities. The disease had originated about 20 years ago, from exposure to cold and damp; and the patient, a clergyman, had tried a host of remedies, prescribed by many of the most eminent medical men of this metropolis, with very little advantage. The nerves chiefly involved, appear to be the superficial branches of the anterior crural and fibular trunks. The results of his experience of the veratria ointment are, that whenever he is able to induce its peculiar effect upon the parts, the pain begins to abate, and then gradually subsides. He is also of opinion, that the recurrence of the paroxysms has not been so frequent.

The fifth case was in some respects similar to the preceding one, but much more severe. The trunk of the sciatic and the branches of the gluteal are chiefly affected.

Frictions with strong veratria ointment (℈ij. to the ℥j.) were repeatedly tried, but without any avail; the sense of burning or tingling could not be induced. Under these circumstances Dr. T. says, that we have no reason to expect any benefit. Whoever therefore gives a trial to his remedy ought carefully to attend to the test which he has pointed out as indicative of the operation of the drug. Three cases of heart affection, in which Dr. Turnbull thinks the veratria has been of decided advantage, have been submitted to our inspection; but the data which have hitherto been furnished, are as yet quite insufficient to warrant us in affixing the stamp of our testimony to the correctness of his opinion. The patients indeed confessed that the cardiac distress was always relieved whenever the effects of the veratria were induced. We willingly give credit to this; because we are satisfied that the medicine exerts a very peculiar effect as a counter-irritant; and it seems to differ from almost all others in this respect, that its operation is confined solely to the nerves of the part, the bloodvessels being scarcely affected.

It is therefore our decided opinion that veratria is a useful and very potent medicine, in certain nervous affections, and that it deserves to be, and no doubt will become an established member of the Materia Medica. Few, perhaps, can hope to obtain such wonder-working results as Dr. T. has had the good luck to achieve. Parents are naturally over-fond of their offspring!

As sincere friends of the profession, we deprecate alike the extremes of indiscriminate applause and of incredulous condemnation.

---

Mr. Cusack on certain Nervous Affections, occurring chiefly in Females.

In our Dublin contemporary for May last, Mr. C. has related some cases of a peculiar form of nervous disease, of which we shall here take a concise notice.

*Case* 1. Mrs. ———, six months pregnant, solicited our author, by letter, to prescribe for a pain in her right side, from which she was never free, and describing it as dull in general, but sometimes acute, and shooting towards her shoulder. She had applied leeches, and used mercury, besides cupping and blistering, without relief. These considerations induced Mr. C. to view the complaint as neuralgic, rather than inflammatory, and he prescribed generous diet, with galbanum and blue pill, followed by morning aperients. The pain ceased after a few doses of the pills, the draughts not having been persisted in, as they disagreed. Four years have now elapsed since the first application, and she always finds relief from the pills.

*Case* 2. Mrs. ——— requested our author to prescribe for one of her domestics, who had fallen into a low state of despondency—was incapable of exertion—had a pain in her right side, and constant palpitation. Tea and coffee were prohibited—regular exercise enjoined, and the following pills prescribed.

℞. Mass. pil. galb. comp. gr. vij.

Mass. pil. hydrarg. gr. iij. Ft. pil. ij. 3tia quaque nocte sumendæ.

℞. Infus. quassiæ, ℥xij.
Sulph. magnes. ℥jss. Ft. haustus 3tia quaque mane sumendus.

It is quite clear that, in the above prescription, ounces are placed instead of drachms. After some weeks, the female was improved in health, and at the expiration of a year was quite well.

A case of pain in the region of the liver, with palpitation of the heart, is briefly alluded to, and where carb. ferri and valerian were successful. In a fourth case there was distressing pain in the right side, between the ribs and the crista ilii, with frequent headaches. It disappeared by taking ten grains of carb. ferri, a scruple of valerian and five grains of soda three times a-day. She derives great advantage also, from the galbanum and the blue-pill, with bitter aperient infusions.

In a fifth case, a poor dispensary female, of intemperate habits, there was great pain on the right side of the spine, between the ribs and crista ilii, which resisted the usual modes of treatment. She was cured by 50 drops of muriated tincture of iron, thrice a-day, in a glass of valerian infusion.

The sixth instance adduced was that of a young girl, who had severe headache of some months' duration, resisting aperients, issues on the crown of the head, &c. She was cured by small doses of blue-pill, with galbanum, quassia, and sulphate of magnesia.

The foregoing cases, selected from a great number, observes our author, exemplify diseases of a nervous character, amenable to mild treatment, but aggravated by severe measures. The hypochondriac pains are frequently considered as indicative of inflammation of the subjacent viscera, and treated by bleeding, mercury, purgatives, &c. by which the unfortunate sufferers are rendered worse. When the head is affected, the patient is often put to great and unnecessary torture, by setons, blisters, &c. Females are far more liable to these affections than males. Much assistance in forming a diagnosis is to be derived from the habits of the patient. Females confined to the house, at their needle, as milliners in large cities frequently are—who drink much tea, with little relish for other food—when complaining of such affections, may be generally considered as labouring under these nervous and anomalous disorders. Nevertheless, it should never be forgotten that such individuals are liable to inflammatory affections, and that a mistake on the wrong side might be of bad consequences. It will, therefore, be prudent to begin with gently depletory measures, as preparatory to the tonics or sedatives, by which we shall be able to feel our way, and run no risk. We recommend an especial perusal of this highly practical paper to our brethren at large. We have been able to give but a very brief sketch of it here.

---

### Spurious Melanosis of the Lungs.

In No. 559 of the Lancet, Dr. Marshall has published two cases of this kind, which he thinks occur more frequently among coal-miners than to others. We shall abridge some of these cases.

*Case* 1. A man, aged 68, a coal-miner from boyhood, had enjoyed good health till within the last seven years, during which he had cough, occasional dyspnœa, with (latterly) purulent expectoration. He presented all the symptoms of phthisis in March, 1831; and in Dec. of the same year, when he came regularly under Dr. M.'s care, the expectoration was of a deep black colour, resembling printer's ink. The quantity was immense, sometimes amounting to two English pints in the 24 hours. The stethoscope, at this time, indicated well-marked cavernous râle under the left clavicle, with total want of respiratory murmur in the left lung.

He lingered till June, 1833, and was at last carried off by diarrhœa. On examination post-mortem, the glands under the sternum were observed to be of a black colour, and so were the lungs on both sides; but the left lung was greatly disorganized, being converted into an immense cavern, traversed by bands of pulmonary structure. There were several small excavations in the other lung, and the excavations, on both sides, contained considerable quantities of the melanotic matter above-mentioned.

*Case* 2. A collier, aged 62, had been affected for many years with chronic rheumatism, together with occasional attacks of dyspnœa. In January, 1833, he was affected with cough and palpitation, as also dyspnœa, but continued at work till June, when his symptoms became so aggravated, as to compel him to give up labour. Some of his expectoration was collected, and was found to resemble a mixture of lamp-black and mucus. The stethoscope detected mucous and subcrepitating râles pretty generally in the right side. The left was without respiratory murmur. He died in January, 33, with the usual symptoms of phthisis.

*Dissection.* The glands under the sternum were filled with black matter. The *left* lung was converted into one immense cavern, nearly filled with an ink-coloured fluid. The right lung: the greater portion of the right lung was deeply stained with the black infiltration, but a considerable portion of lower part was respirable, and floated in water.

Two other cases are alluded to by our author, which, he has no doubt, were melanotic phthisis, but he had not an opportunity of examining the bodies after death. The analysis of the melanotic matter is promised in another communication.

---

### Case of Fracture of the Neck of the Femur, exterior to the Capsular Ligament of the Joint, where Bony Union was effected. By James Edward, Esq. Surgeon, Forfar.

On the 23d Sept. 1833, Mrs. Addison, aged 76, received on the left groin a stroke from a cow, which knocked her down on her right hip. Immediately thereafter, I was called to attend the patient. After a careful examination, I found that the femur was fractured exterior to the capsular ligament of the joint.

The leg was shorter than the other by nearly three quarters of an inch—the toes were everted—the hip flattened. Distinct crepitus could be felt on rotating the limb, without employing extension; there was also a considerable degree of swelling and tension. She complained of acute pains in the groin and behind the trochanter. The limb was placed in the straight position, according to the method recommended by Desault.

The splints and bandages were taken off at the end of twelve weeks, when firm bony union was found to have taken place. The natural length of the limb was preserved. For several days after the accident, smart febrile excitement continued, requiring diaphoretics and opiates. Acute pain in the hip and groin was complained of for several weeks. These symptoms, however, gradually disappeared, after which she became easy and comfortable. Her general health did not suffer much from the long confinement. This case proved more than usually successful, for in six months after the accident she could walk with perfect ease, without the assistance of crutches.

---

### Dropsy cured after Tapping.*

"I had lately an opportunity of satisfying myself as to the recovery of Lieut. G. whose case is adverted to in the 39th Number of the Medico-Chirurgical Review, p. 177, under the head of—'Ascites apparently cured, after Paracentesis Abdominis had been performed 12 times.' He told me he had been in London some time previously, when he had been seen by Sir William Burnett, as well as yourself, and that, after an examination, you had pronounced him cured. As I have seen him at a later period quite well, and it is upwards of eight months since he left this hospital, he may now be considered as completely recovered. I am not aware of there being any similar case on record, except the one I then alluded to, of 'a patient cured after twelve operations,' adduced by the late Dr. Good in his System of Nosology, p. 438.

I have since had another officer under my care, Mr. M. aged 34, affected with general dropsy in a still greater degree, who has been discharged also with a very fair prospect of ultimate recovery (subject of course to contingencies), after double the quantity of fluid measured at any operation in the preceding case, had been twice removed by paracentesis. When admitted, on the 9th of January last, the abdomen was enormously distended—the fluctuation distinct—the lower extremities anasarcous, with pain and much tenderness in the region of the liver and spleen—occasional vomiting of a thick, chocolate-coloured fluid—and great debility. *Paracentesis abdominis* was performed on the 22d of the same month, with great relief, not fewer than 26 pints of water having been drawn off; and it was repeated on the 11th of February, when a similar quantity was again abstracted. After the second operation, the powers of medicine succeeded in preventing the re-accumulation of fluid in the belly, and in effecting the absorption of the cellular effusion in the lower extremities, &c. and when he was discharged, for the benefit of change of air, on the 12th inst. the abdomen and limbs, with the exception of a little swelling and hardness above the right ankle, were reduced to nearly their natural size, and his general health was greatly improved."

We saw Lieut. G. in London, and every vestige of his complaint, as far as a careful examination could determine, had been removed. If the second case mentioned by our friend, Dr. Dickson, should turn out to be as successful as the first, Dr. D. will have been much more fortunate than his neighbours in general.

Dr. D. informs us that he met with a well-marked case of phlegmasia dolens lately in an unmarried female. He also states that he has been making some trials of the veratria in different

* Extract of a letter from Dr. Dickson, Physician of Plymouth Hospital, to Dr. Johnson.

affections; and concludes with these words:—"In some cases, it seems to have great power—in others, none."

---

THE PRINCIPLES OF PHYSIOLOGY, APPLIED TO THE PRESERVATION OF HEALTH, AND TO THE IMPROVEMENT OF PHYSICAL AND MENTAL EDUCATION. By ANDREW COMBE, M.D. Octavo, pp. 320. London and Ed. 1834.

This little work, though not designed for the medical profession, may prove very useful to the medical student—perhaps to many medical practitioners. Be that as it may, it is calculated to prove of eminent service to the reading and more intelligent portions of the public at large. Dr. C. has introduced just as much of descriptive anatomy and physiology as may be usefully applied to the preservation of health, and the improvement of physical and mental education. Most writers on hygiene, at least in this country, have too much neglected the intimate relation that exists between our moral and our physical nature—and how much the cultivation of the mind is connected with organization of body. It is necessary, therefore to study the peculiarities of the corporeal constitution, in order to chalk out and direct the training of the mind. We see that, by attention to this subject, we can develop the powers in general, and those even of specific organs, in the lower animals, with surprising success. The same rules, in all probability, would apply in the human species. In a late work by Prince Puckler Muskau (though we have no very great opinion of this personage on many points) shews the *moral* effects of military drilling in Prussia. The following passage is put into the mouth of one of the Landwehr of Germany.

"Nobody has more reason to acknowledge this than we landed proprietors, who are surprised to find, in the brave citizen soldiers, the only element of that spirit of subordination and docility which is so nearly extinct in our times. I am satisfied that next to the love, founded on reverence, which our people cherish for their king, we have to thank our military system, that amidst the universal unquietness of Europe, Prussia (by no means from want of diffused intelligence,* like Austria, but in the midst of it) has remained tranquil, sedate, and free from all the violent symptoms of modern thirst after mere change. It was thought that to make the whole population soldiers would be to make them ungovernable. The very contrary is the case—this organization has given them habits of order, discipline, and obedience."†

Dr. C. complains, and justly, too, of the little regard which is paid, in medical schools in this country, to the principles of hygiene, or a knowledge of those causes which modify the functions of our organs in health, and consequently predispose, more or less directly, to diseases. Medical pupils and medical teachers are so intent on the description and treatment of diseases, that they are almost entirely occupied with those subjects, to the neglect of hygiene—or, indeed, of physiology.

But to return to our author. We can only glance cursorily at two or three chapters, out of the nine into which the work is divided—though we certainly recommend the perusal of the whole of the book to the generality of our readers, assuring them that, notwithstanding the pretty full occupation of our time in various avocations, we have managed to read the whole of the publication.

Passing over two or three chapters, in which occur many remarks, important even to experienced practitioners—and especially on the sympathy or connexion between the external surface of the body and the internal organs, we shall halt for a moment on the subject

---

* "Light, or intelligence *(aufklärung)*, in the sense in which I here use it, is merely relative, denoting only an extended horizon; it is far enough from *wisdom*, which has generally a tranquillizing, whereas this has a disquieting tendency."

† Tutti Frutti, Stutgard, 1834.

of flannel. Dr. C. appears to attach immense importance to this article of clothing, and there is no doubt but that it is a very great preservative of health, in this and many other climates. Dr. C. condemns the custom of protracting the adoption of flannel till near the approach of Winter, considering it to be as necessary in the Autumn as in January or February. He quotes Capt. Murray, of H.M.S. Valorous, in favour of flannel in a tropical climate. That officer had been two years on the coast of Labrador, among the icebergs; and, on his return to England, was suddenly ordered, with his ship and crew, to the West Indies. He directed the Purser to draw two extra flannel shirts and drawers for each man, and instituted a daily inspection to see that they were worn. He proceeded with his crew of 150 men to his station, visiting most of the West India Islands, and many in the Gulf of Mexico. He returned to England without the loss of a man. Dr. C. does not state how long this cruise lasted; but he attributes this happy result chiefly to the use of flannel. Dr. C. in another place, guards us against making use of insulated facts, as contradicting general principles. We agree with him; but neither should a few cases of the above, or of any other kind, be too much relied on in support of these said general principles. If Dr. C. had ever broiled under a tropical sun, he would have remembered the almost insupportable exhaustion of flannel, and he would probably have advocated the use of calico, which has most of the good properties of flannel, without its heat and weight. Nevertheless, the whole work is written with great judgment and good sense. It is, however, impossible for us to give an analysis of it, on account of its popular tendency. An extract or two will suffice as specimens. One of the most instructive chapters in the work is on the effects of intellectual exercise, moderate and immoderate. There is great truth in the following passage.

"Nervous disease from excessive mental labour and exaltation of feeling sometimes shews itself in another form. From neglecting proper intervals of rest, the vascular excitement of the brain, which always accompanies activity of mind, has never time to subside, and a restless irritability of temper and disposition comes on, attended with sleeplessness and anxiety, for which no external cause can be assigned. The symptoms gradually become aggravated, the digestive functions give way, nutrition is impaired, and a sense of wretchedness is constantly present, which often leads to attempts at suicide. While all this is going on, however, the patient will talk or transact business with perfect propriety and accuracy, and no stranger could tell that any thing ails him. But in his intercourse with his intimate friends or physician, the havoc made upon the mind becomes apparent; and, if not speedily arrested, it soon terminates, according to the constitution and circumstances of the individual case, in derangement, palsy, apoplexy, fever, suicide, or permanent weakness.

As age advances, moderation in mental exertion becomes still more necessary than in early or mature years. Scipion Pinel, in adverting to the evil consequences of excessive moral or intellectual excitement, acutely remarks, that while in youth and manhood the wear of the brain thus induced may be repaired, no such salutary result follows over-exertion in the decline of life: '*what is lost then is lost for ever.*' At that period, we must learn to wait for what the brain is willing to give, and allow it to work at its own time: *to attempt to force it is to weaken it to no purpose;* it becomes excited and quickly exhausted when forced to vigorous thinking.'—'Men of exalted intellect perish by their brains, and such is the noble end of those whose genius procures for them that immortality which so many ardently desire.'*

Who can peruse these lines without the fate of Scott instantly occurring to his mind as a practical illustration of their truth? In the vigour of manhood, few ever wrote so much, or with greater

* Physiologie de l'Homme Aliéné, p. 177.

case. But when, on the verge of old age, adversity forced him to unparalleled exertion, the organic waste could no longer be repaired, and perseverance only 'weakened the brain to no purpose,' till morbid irritability became the substitute of healthy power, and he perished by that brain which had served him so faithfully and so efficiently, but which could no longer withstand the gigantic efforts which he continued to demand from it."

We apprehend that it was more the anxiety of mind than the unparalleled exertions which destroyed the immortal Wizard of the North. It is highly probable that, had his circumstances continued prosperous, his desire of fame, and the pleasure of intellectual employment, would have led him to equal, though voluntary exertions. But then he would not have worked "invita Minerva." Every page which he penned would have been a banquet to himself as well as to his future readers. Adversity affects not only the stomach, but the brain and nervous system generally. The stomach, in fact, is only affected secondarily, and through the influence of the brain. No wonder then that excessive cerebral excitement leads to disease. Pinel mentions the case of a young man, distinguished for his talents, and especially for his chemical researches, who was occupied with a discovery which he hoped would lead him to fame and fortune. To accomplish this object the sooner, he shut himself up in his laboratory for several successive days, taking a variety of stimulants to banish sleep, and keep his vital powers up to the level of the necessary exertions. At the end of eight days he was seized with furious delirium, followed by a regular attack of mania. Tissot remarked long ago that the incessant contemplation of a single object is more injurious than when the objects of study are diversified. In this case, he adds, "there is *only one* part of the sensorium acted upon, and that is kept always on the stretch; it is not relieved by the action of the other parts, and therefore is sooner fatigued and injured—the same rule holding with the brain as with the muscles, that the exercise which, if divided amongst the different parts of which (the brain) it is composed, will strengthen them, will, if confined to a few, exhaust and impair them." This argument is not affected by the well-known law that regular and daily exercise of any one muscle or class of muscles will strengthen that muscle or class of muscles beyond its neighbours. It is, no doubt, the same with the brain, or the faculties of the mind. Regular attention to any one subject of study, will augment the power of that part of the brain concerned in the faculty so exercised.—There is no question that immense mischief every day results to literary and public men—to students, and to people in business from inordinate exertion of the mental faculties. Sir Humphrey Davy and Sir Walter Scott are recent examples. It is said that Newton's mind was, for a time, disordered by excessive application. The premature extinction of early prodigies of genius, as they are called, is generally traceable to the same cause.

"The wonder excited by their performances stimulates them to incessant and severe exertion, unrelieved either by adequate repose or by variety of pursuit; and the exhausted brain either sinks at the period of growth, or becomes so much weakened as to be unfit for the same splendour of manifestations. The more limited the sphere of talent, the greater the danger of its being over-exercised; and hence the frequency of nervous affections in musicians, and others who dedicate their lives to the exclusive cultivation of their arts. It is said that Gretry not only ruined his own health, but lost three highly-gifted and beautiful daughters in succession, from over-excitement of the nervous system thus induced; and there can be no doubt that the melancholy fate of Weber was greatly hastened by intense application. He continued deeply engaged in musical composition long after his health was undermined; and, even when the hand of death was almost upon him, his avocations pressed so heavily that he could not help exclaiming, "*Would that I were a tailor, for then I should have a*

*Sunday's holiday!'* The philanthropic physician will rather be inclined to exclaim, 'Would that mankind would study their bodily structure and functions, and thus learn to preserve longer the health and existence of those whose genius is the source of so many pleasures to the world at large!'"

We could fill many pages with melancholy instances that have come within the range of our own observation, where life has been curtailed—where health has been ruined—and where prospects have been blasted by premature, and we might say preternatural efforts of the young mind, to outstrip their contemporaries in the march of intellect. But we have exceeded our limits, and must conclude with the following short extract, containing excellent advice—too seldom followed by the votaries of science and literature.

"The time best adapted for mental exertion falls next to be considered. Nature has allotted the darkness of night for repose, and the restoration, by sleep, of the exhausted energies of mind and body. If study or composition be ardently engaged in towards that period of the day, the increased action in the brain, which always accompanies activity of mind, requires a long time to subside; and, if the individual be at all of an irritable habit of body, he will be sleepless for hours after going to bed, or perhaps be tormented by unpleasant dreams. If, notwithstanding, the practice be continued, the want of refreshing repose will ultimately induce a state of morbid irritability of the nervous system, not far distant from insanity. It is therefore of great advantage to engage in severer studies early in the day, and devote the two or three hours which precede bed-time to lighter reading, music, or amusing conversation. The vascular excitement previously induced in the head by study has then time to subside, and sound refreshing sleep is much more certainly obtained. This rule is of great consequence to those who are obliged to undergo much mental labour."

We have acknowledged that the foregoing advice is good, and often have we attempted to follow it—without success!

The quietude of the midnight hour, when bustling and noisy servants are sunk in oblivious repose—when messages are no longer coming in, nor footmen waiting for answers to notes, invites to meditation and reflection—and is often the only time which a medical practitioner has for holding converse with his professional brethren through the medium of books. On the other hand, the clattering cook, the meddling housemaid, and the tyrant butler, keep every part of the house in agitation and tumult for hours in the mornings:—add to which, the cold room and half-lighted fire, which the servants are sure to keep in the most uncomfortable state for a troublesome master, who comes among them to disturb the morning operations, are not circumstances particularly adapted to contemplation and study in Winter—while the beauty of a Summer's morning tempt one to go out and inhale the morning air, rather than pore over musty books and thrice-told tales. Such have been among the reasons, or, more properly speaking, the excuses for admiring the precept contained in the foregoing extract, while we pursued a course diametrically opposite!—

> Video meliora proboque—

> Deteriora sequor.

We hope and trust that Dr. Combe's work will be widely diffused among society at large, and that the short and imperfect notice which we have here taken of it, will induce our professional brethren to extend to it their patronage and recommendation.

---

### Liverpool Medical Society—Royal Institution.*

At a meeting of the Liverpool Medical Society, (20th March, 1833,) Dr. Reynolds related instances of the separa-

* Occasional extracts from the minutes of "public business," from 20th March, 1833, till 19th March, 1834.

tion of the pelvis at the *symphysis pubis* in Guinea-pigs during parturition; and wished to ascertain whether it was not the case also in women, at that period? The Society was of opinion that such a separation occasionally took place in women, but was referable to disease.

Dr. Reynolds next stated, that he used croton oil externally, as a counter-irritant, in four or five cases, with great effect. In one instance where it was employed, erysipelas of the face supervened. Dr. R. understood that a similar effect had been produced by the remedy, (related in the Lancet,) and wished to know if any member had used croton oil externally, and with what effect? Drs. Carson and Macrorie had used it externally—the effect was purgative. Mr. Van Oven considered it a valuable counter-irritant, and ought not to be undervalued in consequence of erysipelatous inflammation having been produced in the face. He was of opinion, that any irritant applied to a distant part might, in some cases, produce this effect: it occurred, he thought, when the system was under some peculiar influence. Mr. Van Oven related a case wherein he had applied a plaster of soap cerate and ammonia to a diseased bursa in the knee, in this case erysipelas of the face was the result. Three weeks after the disappearance of the inflammation, again applied the plaster to the knee, and erysipelas of the face again was the consequence.

---

17th April. Dr. Banning in the chair. Mr. Banner related the case of a child, aged 22 months, which had been kicked by a horse on the right parietal bone; fracture with depression, and a slight wound through the scalp, resulted.—Not one bad symptom observed till the 8th day, when there was puffiness of the scalp over the fracture, an offensive discharge, with feverishness. A crucial incision was made, and exposed a triangular fracture, an inch and a half in extent each way. A brain-like substance exuded from the fissure. In a few days, the denuded bone assumed a very unhealthy appearance: the discharge was very offensive. The whole depressed portion of bone was in consequence removed, when *the dura mater was found perforated in three places.* It was now a month since the accident, and no bad symptom has appeared; the wound is nearly healed, and the child quite well in health.

---

13th November. Dr. Baird related a case, illustrative of the difficulty in diagnosis. He had been called to a married woman, aged 23 years, who complained of great pain at the pit of the stomach, and over the region of the heart. Countenance pale; lips bloodless. Tongue white and moist; pulse 140; breathing weak and oppressed. Tendency to syncope on raising the head. The stethoscope indicated the heart's action as peculiar, "after apparently giving a bound, it was succeeded by a number of rapid beats, which might be compared to a tapping against the diaphragm." Complaint of five weeks' standing. Dr. B., from the unfavourable state in which he found the patient, expressed his opinion, that she would not survive many hours. The opinion of members present, as to the nature of the disease, was requested, before detailing the treatment adopted. "Was it a case of pericarditis, with effusion; or adhesion?"

Several opinions were hazarded, as to the case. Dr. B. concluded by observing, that after prescribing the following pill, taken every two hours, the interval between the doses being increased until the 5th day, the symptoms gradually disappeared. *Calomel, quinine, and extract of hyosciamus, of each one grain.*

During the Doctor's attendance, the patient proved five months advanced in pregnancy, which accounted for the symptoms, "shewing that they were merely those of hysteria."

Dr. Lane read a paper on "Hypertrophy of the Mamma," and related a case which he had successfully treated by iodine, ergot of rye, and mercury. In the case alluded to, there was total suppression of the menses: the patient had her mouth made sore by mercury; the iodine was given till the throat became affected; the ergot of rye given in

ʒss. doses, thrice daily for 13 days, when there was a slight discharge, lasting for a few hours. The ergot continued three weeks, with the result of another discharge of nearly a day's continuance, after which, up to this date, the patient had a periodical recurrence of the catamenial issue. Her breasts, too, now were reduced more than one half their original size.

---

27th. Nov. Dr. Carson in the Chair. Mr. Neill related an experiment which he had performed, which shewed, that the optic nerve was permeable by light. Mr. N. said, that if an entire eye (of a bullock or sheep) with the optic nerve divided, within one-eighth of an inch from its entrance into the eyeball, were held up with the pupil towards a candle, the light would be distinctly seen to permeate the optic nerve—its section forming a luminous spot, circular in shape. Some discussion arose upon this experiment, when the President recommended, that the members of the Society should repeat the experiment for their individual satisfaction. Mr. Banner read a paper "On the Circulation of the Blood in the last stage of Epidemic Cholera," in which he endeavoured to explain the integrity of the mental faculties— and the absence of mortification of the extremities. The former he accounted for by supposing that the bloodvessels of the brain were kept in the state of distention necessary to the discharge of the functions, by the stagnation of the blood (caused by its increased thickness) impeding its return to the heart. The rare occurrence of mortification of the extremities was accounted for by the fact (?) that during respiration the usual quantity of oxygen was not absorbed, in consequence of which carbon was superabundant in the blood, and acted as an antiseptic. The coldness of the extremities Mr. B. sought to explain by the non-absorption of oxygen preventing the evolution of caloric.

Mr. B.'s views as to the absence of mortification were combated by several members. With respect to the cause of the mental faculties continuing unimpaired—Dr. Carson, (senior) thought that it depended on the peculiarity of the circulation within the cranium, the vessels there being enabled to retain their contents in consequence of their exemption from the direct influence of atmospheric pressure.

---

11th Dec. Mr. Christian in the Chair. Dr. Anderson related a case of inguinal hernia, which he had treated by the recumbent posture, as recommended in a number of the *Archives Generales de Medecine*, for 1831. The patient was a woman, 35 years of age, who after an accession of grief, nine months previously, observed a tumor in the inguinal region, the size of a duck's egg. When Dr. A. saw the patient, the tumor pressed down with great force. The woman was placed in the recumbent posture, and the cure was effected in the shortest space of time indicated (in the foreign periodical referred to)—viz. 31 days. At the expiration of a week, the orifice, which at first admitted the finger freely, was much contracted; and at the end of the month there was no protrusion whatever on coughing—the patient resting on her knees. Three weeks had elapsed since the cure, and the orifice does not now admit even the point of the finger.

Dr. Scott read a paper "On the effect of Sleep upon the Vital Functions," which was classical and philosophical in the extreme, after the discussion of which, the Society adjourned.

---

23d Dec. Dr. Lane in the Chair. Mr. Neill read a paper in which he related several cases of disease of the retina, with double vision; and in which *strychnine*, both externally and internally, had produced beneficial effects. One case was remarkable for having the double image presented to the diseased eye, even when the healthy eye was closed: this case was accompanied with a rushing sound in the ears, relieved by strychnine, of which four grains were taken in eight days. The diplopia was likewise benefited by the treatment. The accidental death of the patient (from a weight falling upon his head) prevented the final result being ascertained. Mr. N. laid it down as an invariable rule that the strychnine would do no good unless the IRIS retained *some* degree of mobility.

---

22d Jan. 1834. Dr. Carson in the Chair. Mr. Windsor enquired whether any of the members present had observed during the epidemic, measles so extensively prevalent of late,—a pustular affection of the lips and inside of the cheeks, and angles of the mouth? In three children of the same family, after going through measles, were, on the authority of a friend, affected with this pustular eruption; which—and this he considered the remarkable feature of the cases—was afterwards *communicated to both parents!* Dr. Carson met with numerous cases of the kind within the last two months, both in adults and in children, apparently quite unconnected with measles. Dr. C. considered it an epidemic affection. When physician to the Workhouse, had seen it prevail extensively among children, and yielded to calomel and scammony purges which brought away *scybalæ.*

Dr. Duncan said, that during the month of Dec. when the epidemic of measles was at its height, a majority of the children whom he saw were affected sometimes *during* the rubeolar eruption, but more frequently after its decline—*with ulceration of the gums of a gangrenous character,* in one or two cases extending backwards to the fauces and terminating fatally. Many children also had a pustular eruption round the mouth and alæ of the nose. He had observed a few cases unconnected with measles.

Mr. Rogerson read his paper "On the necessary relations of Medicine to the State, or on Medical and Hygienic Police," and after a short discussion, the Society adjourned.

---

5th Feb. 1834. Mr. Christian in the Chair.—In connexion with a case before the Society, Mr. Christian observed—that in apoplexy arising from narcotics, emetics were useful. Was called to a patient whom he knew to have been taking *ex. sarsaparillæ,* and whom he found with stertor and dilated pupils, &c. the symptoms in fine of apoplexy. It appeared that the patient had taken ℈ij. or ℈iij. *ext. hyosciami* instead of sarsaparillæ. His pulse was soft and very slow; and recovered in a few hours from using emetics. Mr. C. thought the secretions of stomach sometimes caused symptoms of apoplexy:—had himself for some years been subject to dimness of sight, confusion of the senses, indistinct articulation, &c. followed in about half an hour by sickness, a little sour bitterish water coming up. Ultimately discovered, that the symptoms were owing to coffee or green tea, which he was in the habit of taking:—was completely relieved for some years after giving them up.

Dr. Carson, jun. read a paper on the Hibernation of Animals, in which he maintained, 1st. That all the motions of the body, vital and animal, are produced by impressions conveyed from the brain and spinal marrow through the motor nerves. 2d. That the amount of these motions is proportioned to the degree of excitement of the brain. 3d. That there is a degree of excitement of the brain below which it is unable to produce contractions in the muscles of animal life, and to develop the phenomena of sensibility. 4th. That there are degrees of excitement of the brain at which the several muscles of respiration are in order abstracted from the influence of the brain; and a still lower diminution of this excitement which is followed by a gradual abstraction of the muscles influencing the circulation of the blood, viz. the heart and arteries, from the influence of the nervous centres. 5th. That the degree of excitement of the brain depends on the amount of impressions communicated to it by the sentient nerves. 6th. That in the contact of the extremities of the sentient nerves with external objects, is to be sought the only source of nervous energy. 7th. That the power in these extremities of receiving impressions is susceptible of increase or diminution. 8th. That the power is diminished by long-continued use, and the influence of certain remedies. 9th. That sleep is caused by the exhaustion of the energy of the brain, below that point at which sensibility ceases to exist; which exhaustion arises, 1mo, from the brain continually distributing through the nerves the energy which is requisite to keep up the ordinary actions, *e. g.* the vital actions: 2do, From the diminished power of the sentient extremities to receive impressions *ab externo:* 3tio, The instinct which leads us to abstract the sentient extremities as much as possible from the influence of external agents. 10. That the power of the sentient extremities to receive impressions, is repaired by the vital processes which are continually going on, repairing defects of the frame, and which processes are last of all abstracted from the influence of the brain. 11. That the degree to which sleep may be carried is proportioned to the previous activity of the animal, and its power of abstracting itself from the influence of external impressions. 12. That the muscles of the palate and glottis being earlier abstracted from the influence of the nervous centre than the other muscles of respiration, snoring is a consequence, which, from the uneasiness it excites when carried to a great degree, is *one* of the limits to the degree of sleep. 13. That the muscles of the right side of the heart and the pulmonary capillaries are earlier abstracted from the diminishing influence of the nervous centres than those of the left side; hence, accumulations of blood in the capillaries of the lungs—a *second* limit to the degree of sleep. 14. That did neither of these limits exist, the excitement of the brain might be diminished to such an extent, that the animal actions, and greater part of the vital actions, might cease altogether, and the only processes which might continue be a low degree of the respiratory process, the organs of which are last abstracted from the influence of the brain; and that such is the condition of the hibernating animal.

---

19th Feb. Dr. Carson, sen. in the Chair. Dr. Macintyre had met with a curious case:—Account given by the patient (a workman on the railway was this)—after drinking 2*s.* 6*d.* worth of ale, fell asleep in the open air: on awaking, found something wrong with his penis. On examining, discovered a large iron nut (*female* screw, 11 ozs. weight) behind the glans. Endeavoured in vain to remove the nut, even with a hammer and chisel, at the same time fastening his *penis* in a *vice* (!) Twelve hours after applied to a surgeon; the nut had by this time made its way close upon the pubis—the portion intermediate from glans to nut swollen, and as if gangrenous. The surgeon scarified the *penis*, but, being unable to remove the screw, took the man to an *instrument*-maker, who, after fixing the penis in a vice, and after working for several hours with very fine instruments, succeeded in removing the iron: after a portion being removed, the remainder, shewn to the Society, weighed upwards of nine ounces! Dr. M'I. added, that he had reason to believe the story told by the man was a pure invention; and that the individual himself had caused his misfortune.

Dr. Carson, sen. read his paper—"*On the Powers supplied at the Termination of the extreme Vessels, for maintaining and promoting the Motion of the Blood.*" He stated that the only serious objection made to his theory of the suction powers of the heart, was that advanced by Dr. Arnott: that when applied to draw a fluid through incompressible tubes, such as the veins, its only effect would be, to cause the sides of the vessel to collapse.

The theory proposed in the present paper was chiefly to obviate that objection. Dr. C. said that, as the arteries were destined to supply fresh materials to the system, and to convey blood to the veins, the orifice of an arterial capillary must have two communications—one with the particle deposited for the repair of the system, the other with a venous capillary, directly, or through the medium of cells. In like manner the venous capillary was in communication, 1st, with the excrementitious particle which it was about to absorb; and, 2dly, with an arterial capillary, either directly or through the medium of cells. In this intermediate passage between the arteries and veins, Dr. Carson supposed that particles, on being excreted by the arteries, lost the protection of the vital laws, and, becoming subject to the ordinary laws of dead matter, underwent some chemical change, probably of the nature of fermentation, in virtue of which they became expanded in entering the mouths of the venous capillaries: and that this increased bulk of the particles counteracted the tendency of the suction power of the heart, to close the mouths of the veins, which were thus left constantly open by a fresh succession of expanded particles. Dr. Carson farther contended that, in consequence of the great curvature of the capillaries, they were very incompressible, and being kept always filled, and their sides being prevented from collapsing by the expanded blood, this fluid, unable to retrograde in consequence of the current from behind, was withdrawn in the other direction by the suction of the heart. A swelling tide, in one continued wave, thus advanced from the extreme vessels in the direction of the veins, the vis à tergo sending it forward until it came within the influence of the heart's suction. As the vessels always have some connexions enabling them to preserve their tubular form, the objection, that the power of the heart would tend to collapse their sides, was in a great measure removed. Dr. C. contended, *on the supposition of the present theory being correct*, that *his theory of venous motion was as firmly established as the theory of the tides.*

Discussion ensued on the reading of the above paper, in the course of which several objections were urged by different members. The principal objection, in Dr. C.'s opinion, was one advanced by Mr. Cocks—that, *if Dr. Carson's theory were correct, the specific gravity of venous blood should be less than that of arterial.* Dr. Carson admitted that this suggestion deserved consideration.

---

5th March, 1834. Dr. Reynolds in the Chair. Dr. Baird stated the particulars of an obscure and fatal case in a patient of his—a lady, 57 years of age, temperate, but spare, and highly nervous, who enjoyed good health till Dec. last, when she had an attack of inflammation of lungs. During a convalescence of six weeks, was occasionally affected with blindness and headache. An attack of shivering came on, followed by sweatings, recurring for four days, when Dr. B. was called in. Her state then was, pain in the loins and umbilical region—pulse 100, full—tongue moist, clean at edges, slightly furred in centre, with erect papillæ—no headache—pain of abdomen attributed to the severe operation of purgative medicine. Leeches applied, and pills of calomel, opium, and antimony administered, with decided relief. Next day eyes were injected, temples were leeched—bleeding from profuse, and stopped artificially on the approach of syncope. Pulse up to 140—headache—violent action of carotids. Dr. Baird ordered VS. which again induced syncope. Stupor came on in the evening. Head shaved, cold applications; yet she died comatose, 14 hours after losing blood from the arm. The blood was buffed. No post-mortem examination procured. What was the nature of the disease? and had an abscess formed in the brain, or was there serous effusion?

Mr. Banner thought the disease *neuralgia*: thought the depleting measures used had hastened her death. Mr. Neill thought that, from the affection of the sclerotic coat, pain in the loins, and the success of the treatment, V.S. excepted, shewed the disease to be of a rheumatic nature.

Dr. Duncan was unable to draw any other inference from the suite of symptoms detailed by Dr. Baird, than that the case was fever. Dr. Bryce thought the brain was organically affected. [Dr. Baird had omitted a most important circumstance in narrating the case—"on the day when the leeches were applied to the temples, the patient *had a fit resembling epilepsy, and one arm was spasmodically contracted.*" This was communicated *after* the Society adjourned, to the secretary, Dr. Duncan.]

Dr. Thorburn called the attention of the society to the frequency of deaths which had lately been observed among the aged and infirm. This was the case in some districts of Scotland, where no epidemic was prevalent, but where the season had been unusually variable. Dr. T. wished to know whether the same observation had been made in Liverpool? Dr. Anderson said that, so far as he had observed, old people had, during this Winter, been unusually free from the diseases to which they were generally subject during that season of the year. Dr. Macintire thought the subject had better be discussed after the publication of the bills of mortality.

Dr. Edwards had been lately called to a female in labour, and found every thing natural; os uteri dilated—membranes protruding—the *head* presenting. The pains being sluggish, desired his patient to get out of bed, and walk about the room. She did so. In a short time, "waters" came away—"pains" strong. Dr. E. made a second examination, and found, to his surprise, a *hand* presenting! It was necessary to turn the child, which he effected; the child's life being with difficulty preserved. As Dr. E. was confident that the *head* presented on the first examination, he suggested the necessity of extreme caution in allowing the woman to leave her bed after labour has commenced.

Dr. Bryce said it was the first time he had ever heard of a woman being desired to get out of bed under similar circumstances. Drs. Thorburn and Anderson, Mr. Rogerson, and other members, thought the course pursued by Dr. Edwards, was not only usual, but what was recommended by lecturers, and authors on midwifery. Mr. M'Kee, who had delivered many thousand women, concurred in the observation just made, but doubted if necessity existed for turning the child. In the course of an extensive practice, had never found it necessary to resort to the operation of "turning," in cases of *hand* presentation.

Dr. Thorburn read a paper, entitled "*Notes of an interesting Medico-legal Case of Succession, decided at Edinburgh in* 1833," submitted to the Society with the view of eliciting discussion—whether or not the test required, agreeably to the law of Scotland, as to *the state necessary to enable the new-born infant to inherit*, be not still the most eligible and equitable of any which, in the present state of medical science, could be substituted? An animated and protracted discussion ensued, after which the Society adjourned, Dr. Bryce having previously announced for next evening—a paper "on Diseases usually styled Functional." J. S. T.

---

The Transactions of the Provincial Medical and Surgical Association—Vol. II.

This volume arrived too late in the quarter to admit of notice in the Review department. We have only been enabled to cast a superficial glance upon its contents; but we think we have seen enough to pronounce it not inferior to its predecessor—the first volume. It must prove a source of high gratification to every lover of our science, to observe the rapid strides in intelligence, energy, and action, which provincial surgeons and physicians have made. Their hospitals and infirmaries are provided with excellent medical schools—their literary and scientific societies are well organized and well attended—and their published transactions now convey to the world the facts which they observe and the experience they acquire. We of the metropolis hail this advance with feelings of satisfaction and delight—we stretch our hands to our country brethren, and greet them as worthy competitors in the race. May they persevere, and shew the world the universality of British industry and information.

We shall take this opportunity of singling out some of the shorter papers contained in the present volume, and defer the consideration of the rest till our next. It might appear invidious did we mention individuals, when the mass of the contributors are so respectable; yet we hope that their well-earned reputation may warrant our particularizing Dr. Forbes, of Chichester—Dr. Barlow, of Bath—Dr. Bardsley, of Manchester—Dr. Brown, of Sunderland—and Dr. Conolly, of Warwick, as authors of valuable memoirs and essays, or reporters of interesting facts.

There are three topographical articles, occupying one hundred and fifty pages—a well-written biographical memoir of the late Dr. Darwall of Birmingham—an article on medical jurisprudence—two infirmary reports—and fourteen miscellaneous papers, chiefly occupied with the details of facts.

Our selections in the present instance will be guided, as we mentioned, by the brevity, rather than the value of the contributions. Those which we propose to notice are—the history of a case of lithotomy by the rectum—a case of hydrophobia—a case of tuberculous affection of the right kidney—a case of uterine hydatids—a case of foreign body found in the heart of a boy. Our next Number will contain an analysis or critique on such of the remaining papers as appear adapted for those purposes.

I. CASE OF LITHOTOMY BY THE RECTUM. By JAMES DAWSON, Esq. Surgeon to the Liverpool Infirmary.

Robert Cox, æt. 3½, sickly and diminutive, was admitted into the Liverpool Infirmary in Jan. 1822.

He had laboured for six months under symptoms of stone in the bladder. The bony outlet of the pelvis was deformed, the space between the conjoined pudic and ischiatic branches not exceeding one-third of an inch. The course of these branches, to within one quarter of an inch in front of the anus, was nearly parallel; from this point to the tuberosities, their divergence was abrupt, leaving ample space for the anus itself. The urethra was found to be short and very narrow, admitting with difficulty the smallest instrument, every attempt at introducing which was attended with rigors, nausea, and complete prostration of strength. The act of micturition itself was followed by a state approaching to syncope, and by prolapsus ani.

No stone could be detected by the most careful examination, and the boy, after remaining for a few weeks in the infirmary, was sent home. In five months he returned, worse in every respect.

Mr. Dawson now discovered, on passing his finger into the rectum, that about half an inch above the sphincter ani, on the anterior aspect of the gut, a bulging of the membrane existed, and that through it a foreign body, apparently about the size of a large Spanish olive, could be felt. On sounding the bladder, the instrument struck against a stone, which lay in this situation.

The child's health was improved by appropriate means. The irritability of the bladder was relieved by the regular introduction of a gum catheter, and enemata of cold water quieted the mucous membrane of the rectum. In Jan. 1833, he was deemed to have arrived at a proper state for the operation, which was thus performed.

"He was bound by no ligatures. The nurse supported him on a pillow on her lap, in the usual position. A gum lancet, having its anterior edge rounded and very keen, was laid flat on the finger, which, thus armed, and oiled, was introduced through the anus, so as to reach a point a short distance beyond the recto-vesical pouch, when its edge was turned upwards, and a decided cut made, by drawing the instrument from behind, forwards, in the median line, through the walls of the pouch, and up to the stone, on the hard surface of which, the edge of the lancet was distinctly felt to grate.

I may mention, that the back part of the blade of the instrument was blunted, which allowed the point of the finger to project beyond it, and which was thus at liberty to direct, as well as to execute, the intended incision. After pausing a few moments, to allow time for the retraction of the divided structures, the finger was again passed, when the calculus was felt to be entangled among a mesh of elastic fibres; hence a second section became necessary, in effecting which the speculum was employed.*

* "The employment of this instrument was productive of great torture to the child, and as the degree of stretching to which the mucous membrane was exposed by its presence, was undesirable in every point of view, it was instantly withdrawn."

The calculus was now found seated in the upper, or vesical region of the sac, whence, having been displaced by the finger, it fell into the rectum, from among the valvular folds of which, after eluding the attempt once or twice, it was finally withdrawn by the help of Pellier's double silver wire, which served the purpose of scoop and lever. No blood was lost. The operation lasted nearly five minutes.

The calculus was found to be much smaller than we had been led to expect. I have omitted to state that, during the operation, a quantity of hardened or dried mucus escaped along with the fæces, through the sphincter ani; it resembled, in form, the peeled skin of a small apple, and it struck us, at the moment, that this substance might have been coiled round the lower moiety of the concretion, something after the manner of the cup of the acorn, and might, in this way, have added to its apparent magnitude."

We need not mention the subsequent particulars further than by stating that, on the tenth day after the operation the urine passed in a full stream through the penis, and was never afterwards detected in the alvine evacuations. The child remained in the Infirmary for six months, when he was dismissed. Soon afterwards he died of the epidemic cholera.

It is clear that the operation was adapted to the circumstances of the case, and it is satisfactory that no fistulous communication remained between the rectum and the bladder. Probably the sacculated condition of the latter was favourable to the non-occurrence of fistula. We might take the liberty of hinting to Mr. Dawson the value of literary compression. The expansive force of narration is great, and requires resolute resistance. The original case is diffused through ten pages.

II. Case of Hydrophobia. By R. Grinrod, Esq. of Manchester.

The details of this case, which are given at full length, do not seem to require particular notice. We may simply observe that the patient, a boy, was six years and a half old—that he was bitten over the left eye by a rabid dog on the morning of Dec. 2d, 1833—that the parents immediately washed the wound, and took him to a public institution, "where, however, no decisive measures were pursued,"*—that Mr. Grinrod, hearing of the circumstance on the 4th inst. excised the parts and afterwards applied a strong solution of caustic—that the wound soon healed—and that the symptoms of the disease commenced by an aversion to drink water on the 8th of January.

On the 11th of January he died.

*Dissection.* The supra-orbital and nasal branches of the fifth pair of nerves, which supplied the parts bitten by the dog, were apparently healthy. No morbid appearances were detected in the brain, nor in its membranes, nor in the spinal chord.

The heart was remarkably firm and contracted, and contained no blood. The lungs were of a dark colour in some parts, and in others more of a rosy hue. They crepitated as usual, and did not appear to contain mucus. There was no fluid either in the pericardium or the cavity of the chest. The mucous membrane of the trachea, immediately before its bifurcation, was highly vascular. The submaxillary and sublingual glands were much enlarged.

On opening the pharynx, it was evident that this part had been much affected during life. The greater portion of its inner suaface was intensely inflamed, and terminated in a defined line opposite the cricoid cartilages. The velum pendulum palati, uvula, and most parts of the pharynx, shewed considerable development of the mucous glands. The tonsils were slightly enlarged. The œsophagus was greatly inflamed near its termination, and, in one part the mucous membrane was slightly abraded. The whole mucous membrane was so

* We could almost wish that the name of the institution, and the parties concerned, were published. Surely there was culpable and shocking carelessness.

softened, that the slightest touch of the scalpel separated it.

The stomach was remarkably contracted and completely empty. Intense inflammatory patches were seen on the rugæ, which were very much developed. The mucus was very thick, and had, in some parts, an unusually deep colour. On one of the rugæ was observed a dark spot, about the size of a small shot. The stomach appeared healthy between the rugæ, but had, in some places, a darker colour than natural. The mucous membrane of the duodenum displayed intense inflammation also, and the glandulæ solitariæ were exceedingly enlarged and very numerous. The bowels were not particularly examined. The liver was healthy. The gall-bladder contained bile, and the urinary bladder about an ounce and a half of urine. Kidneys apparently healthy, as, also, the spleen. The whole of the nervous system was remarkably developed.

The par vagum, glosso-pharyngeal, gustatory, pharyngeal, and phrenic nerves, were in a healthy condition. The ganglia of the sympathetic were larger, and appeared of a deeper colour than usual.

Mr. Grinrod adds in an Appendix some particulars respecting the dog, which he had had an opportunity of watching for several hours previous to its death. It was a bitch, about two years and a half old, and was at heat for three or four days previously to her exhibiting symptoms of madness. She was examined, after her death, by Mr. Jordan, an anatomical lecturer in Manchester.

"The lungs were of a very rosy colour, but their structure was healthy. The quantity of mucus in the bronchial tubes was small. The mouth healthy, but very parched. The whole of the pharynx was of a deep rosy colour, which terminated in a defined line immediately opposite the cricoid cartilages. The larynx was healthy. A considerable vascularity was manifest at the lower part of the œsophagus, and was continued into the stomach. On opening the stomach, a mass of substance was observed, which had certainly the external appearance of a bird's nest. On being separated, it was found to consist of straw, wood, leather, &c. These materials were in a remarkably dry state. Upon being removed, the mucous membrane was found to be covered, in many parts, with a tough mucus. The rugæ were particularly manifest, and upon their surface were observed, here and there, intense inflammatory patches, and in other parts, black or dark brown streaks, which, in a few hours, became still more perceptible. The external surface of the stomach seemed to be healthy. The par vagum and nerves of the tongue were perfectly healthy. The salivary glands were of the usual colour and size."

Those who are acquainted with recorded cases of hydrophobia will perceive that the appearances found on the dissection of the boy and of the dog, displayed the characters most commonly observed upon similar occasions. Whatever may be the actual nature of the malady, the mucous membrane of the pharynx, œsophagus, and stomach displays the most obvious anatomical alterations. The dread of deglutition is probably dependent upon this condition, and mere reasoning would induce the practitioner to rely most upon remedial measures adapted to remove it. The obvious indications would appear to be local depletion and cauterization, with calomel and opium, a combination which exerts more influence on such kinds of inflammatory action than any other medicinal agent. But experience has proved how inefficient are such theoretical suggestions, and compelled us to acknowledge, that some specific poison introduced into the system defies the powers of what we may term symptomatic treatment.

### III. A Case of Tuberculous Affection of the Right Kidney, with extensive Spinal Disease. By J. Prichard, Esq. of Manchester.

Mademoiselle Le Noir, æt. 68, consulted Mr. Prichard in June, 1828, on account of a very acute pain, referred to the sacrum and to the front and middle of both thighs, especially the right. The pain was nearly constant, and much aggravated by the recumbent posture. There was also considerable pain, on pressure, in the region of the kidneys, and occasional irritation of the bladder, with red sand at times in the urine. The spine was curved, though not angularly. The general health was pretty good.

Previously to 1818, the lady had suffered occasional attacks, which appeared to arise from gall-stones. The commencement of the symptoms described above was in June, 1827, when, after muscular exertion, pain was suddenly felt in the thighs, and shortly afterwards in the sacrum.

Mr. Prichard prescribed some alkaline remedies, and cupped the patient twice on the loins. She then went to town, and from that to Paris, her sufferings being much increased by her journey. In Paris, she voided a large biliary concretion from the rectum. In May, 1829, she returned to Leamington. The pain was very severe, compelling her to remain day and night upon her hands and knees. A considerable angular projection had appeared about the lower dorsal vertebræ. Caustic issues were established in the back. In June she was attacked with fever, and numbness and loss of power in the lower extremities. This latter symptom fluctuated greatly during the succeeding months, and spasms in the limbs were sometimes experienced. From November to February, 1830, there was manifest amendment. In the latter month, a tumour was discovered in the region of both kidneys, that on the right side being circumscribed and more elevated than that upon the left, while on both sides, particularly upon the right, a pulsation synchronous with that at the wrist, could be distinguished. In June, the rectum and bladder became partially paralyzed. In July, pus was discharged from the bladder. On the 20th Sept. the patient died, worn out.

*Dissection.* There was nothing of a very remarkable character about the liver. The gall-bladder was shrunk into the form of a semi-cartilaginous membrane, puckered up into several elevated folds, within one of which a small gall-stone was lodged. The ducts displayed little peculiarity.

"The right kidney, much enlarged, weighing 12 oz. formed the external tumour. Its adipose and cellular investment adhered firmly to it, and the lower and back part of which shewed marks of recent inflammation, being, in its thickened state, inseparable from the organ itself. The renal arteries, veins, and ureter, were healthy, excepting that the latter contained curdy pus. A section of this kidney exposed a mass of extensive disease, the centre of which had a firmly gelatinous character, and was intersected by white bands. In other parts the appearance was tubercular, more or less advanced to a state of *ramollissement;* its superior portion, from which the discharges of curdy purulent matter from the bladder proceeded, was in a state of suppuration."

The left kidney weighed 4 ozs. It had a small hydatid on its surface. The bladder was small, and much thickened.

A considerable protuberance of the spine presented itself, about the last dorsal and first lumbar vertebræ, the spinous processes of which, with that of the second lumbar vertebra, were separated from their bodies, and partook of the morbid changes surrounding them. The first lumbar vertebra was converted into a soft carious mass, having partially the same character of tubercular *ramollissement* as the kidney. The dura mater, in the vicinity of this mass of disease, appeared healthy; on opening it, the medulla spinalis had evident marks of recent inflammation, and the pia mater investing it and the proceeding nerves, were much injected. On removing the psoas muscles, several tumours, as large as a walnut, appeared, closely attached to the spine, two on one side and one on the other, having the same character of scrofulous tubercle as the other diseased parts.

The aorta, where it passed over the protuberance of the spine, adhered firmly to it. The vessel was partially ossified.

Mr. Prichard considers the case as interesting upon two accounts: first, because it shews at what a late period of life scrofulous disease may affect the spinal column; and, secondly, because he thinks the pulsation in the renal tumor was owing to inordinate action of the renal arteries.

It is certainly not common, yet it is not very rare, to see caries of the spine and psoas abscess in persons advanced in life. Our large metropolitan hospitals not unfrequently contain such cases. We confess that we never witnessed pulsation in scrofulous tumors, from augmented action of the vessels supplying them. We have seen pulsating fungoid tumors, as most practitioners possessed of any experience have done. But we do not imply, in opposition to Mr. Prichard, that his was an instance of this description. Yet some of the features wear a resemblance to those of the fungoid disease.

### IV. A CASE OF UTERINE HYDATIDS. By W. WATSON, Esq. of Warwick.

The patient, a female, 22 years of age, had been subject to severe periodical headaches, attended with irregular and violent action of the carotids. The menstruation had never been properly established. The last attack was in the early part of the present year. After it had been subdued by appropriate remedies, an issue was established in the leg. The patient was very delicate. She had been married for ten months.

"On the morning of the 27th of May, 1833, I was again summoned to attend her. She was in bed, and complaining of most violent pain in the bowels, and, in short, nearly all round her body, together with a strong sensation of bearing down. On applying the hand to the abdomen during a paroxysm, I found the uterus firmly contracting, and occupying a space equal to that of a pregnancy of five or six months. There had been a coloured discharge, she then informed me, for the last eight weeks. She had no suspicion of being pregnant, nor was she at all conscious of any increase of size, and appeared not a little surprised when, on my directing her to place her hand upon her bowels, she discovered, for the first time, the increase in volume of the abdomen. Under the impression that she was about to miscarry, I administered an opiate draught, and directed the application of cold externally. She had rigors, with accelerated pulse; a white and clammy tongue; considerable thirst; bowels open. On repeating my visit, in half an hour, I was informed that the draught had been rejected. The uterine action was, nevertheless going on vigorously, and, at the moment of my entering the house, something was expelled from the uterus, which an attendant was proceeding to remove, but, from the unexpected and novel appearance of what presented itself to her view, she withdrew in considerable alarm, and requested my immediate attendance.

The cause of alarm consisted of an enormous growth of hydatids, of the grape form, weighing twenty-six ozs. Very little hæmorrhage followed the expulsion of this mass of hydatids; and, although much relief was obtained by it, it was evident, from the character of the pain which continued, and from the external examination of the uterus, that something more was yet to pass. The immense clusters of grape-like hydatids were connected with, and depending from, a substance of considerable extent, having much the appearance of omentum. The portion which had evidently been in connexion with the uterus appearing particularly solid, I divided it with my scalpel, but it consisted of nothing more than a condensed collection of smaller hydatids, mixed with the substance already alluded to."

In the evening, another large branch of hydatids was passed, after which the pain ceased speedily. Some tenderness on pressure of the abdomen prevailing, leeches and salines were ordered, with relief. In the night of the 28th, she had slight shiverings; next day the mammæ were found to be swollen, and a good deal of milk had escaped. A few detached hydatids were voided. After this, the patient advanced steadily to recovery. The secretion of milk remained for some time. A troublesome sanguineous discharge from the uterus yielded to injections of port wine and water.

The following peculiarities in the case, peculiarities distinguishing it from those displaying the ordinary characters, are succinctly pointed out by Mr. Watson.

"The chief peculiarities of the case before us, consist, first, in the absence of the membranous bag, so strongly insisted upon by some writers, as being *invariably* present. In this instance there was not the slightest appearance of any thing of the kind, neither at the time of expulsion nor subsequently. Neither could it have passed away in a gangrenous state, for during the first weeks after that event, the discharge was so trifling, and, at the same time, so pale, that had any thing of the kind existed and passed, it could not have escaped observation. Secondly, after the expulsion of the first cluster, as well as after the second, no hæmorrhage, possessing, in the most remote degree, a violent character, supervened. On the contrary, the discharge was remarkably small in quantity. It is, also, worthy of remark, that the patient herself was perfectly unconscious of any change in her person, which could lead her to the supposition that she was pregnant; and even at the moment of my first visit, on the morning of the 27th, assured me, as already stated, that she had no reason to suppose any thing of the kind existed."

### V. Singular Case of a Foreign Body found in the Heart of a Boy. By T. Davis, Esq. of Upton-upon-Severn.

We do not know that we can usefully abridge the following particulars.

"On Saturday evening, January the 19th, 1833, I was summoned to attend Wm. Mills, aged 10, living at Boughton, two miles from Upton. When I arrived, his parents informed me that their son had shot himself, with a gun made out of the handle of a telescope toasting-fork. To form the breach of the gun, he had driven a plug of wood, about three inches in lengh, into the handle of the fork. The touch-hole of the gun was made after the charge of powder had been deposited in the hollow part of the handle. The consequence was, that when the gunpowder exploded, it forced the artificial breach, or piece of stick, from the barrel part of the gun with such violence, that it entered the thorax of the boy, on the right side, between the third and fourth ribs, and disappeared. Immediately after the accident, the boy walked home, a distance of about forty yards.

By the time I saw him, he had lost a considerable quantity of blood, and appeared very faint; when I turned him on his right side, a stream of venous blood issued from the orifice, through which the stick entered the thorax. Several hours elapsed before any degree of re-action took place. He complained of no pain.

For the first ten days or a fortnight after the accident, he appeared to be recovering, and once during that time walked into his garden and back, a distance of about eighty yards; and whilst there he amused himself with his flowers, and even stirred the mould. He always said he was well, and was often cheerful, and even merry. There was no peculiar expression of countenance, excepting that his eyes were rather too bright.

After the first fortnight he visibly emaciated, and had frequent rigors, which were always followed by faintness. The pulse was very quick. There was no cough, nor spitting of blood. The secretions were healthy. He had no pain throughout his illness."

He died on the 25th of February, five weeks and two days after the occurrence of the accident.

*Dissection*. On opening the thorax, a small cicatrix was visible between the cartilages of the third and fourth ribs, on the right side, about half an inch from the sternum.

The lungs appeared healthy, with the exception of a small tubercle at the right, and at its root, near to the pulmonary artery, a small blue mark in the cellular tissue, corresponding, in size, with the cicatrix on the parietes of the chest.

Half an ounce of serum was contained in the pericardium.

"When an incision was made into the heart, so as to expose the right auricle and ventricle, we were astonished to find, lodged in that ventricle, the stick which the boy had used as the breach of the gun, the one end of it pressing against the extreme part of the ventricle, near the apex of the heart, and forcing itself between the *columnæ carneæ* and the internal surface of the heart; the other end resting upon the auriculo-ventricular valve, and tearing part of its delicate structure, and being itself encrusted with a thick coagulum, as large as a walnut.

We searched in vain for any wound, either in the heart itself or in the pericardium, by which the stick could have found its way into the ventricle."

Mr. Davis seems puzzled to imagine how the stick got into the ventricle. He supposes that it entered the vena cava, and was carried with the blood into the right side of the heart. The stick would appear to have been about two inches and three quarters in length. There are, therefore, obvious physical difficulties in the way of its getting from the vena cava to the auricle. We know of no better mode of accounting for the fact; and we leave the explanation to such of our readers as can find one.

### VI. METHOD OF REDUCING DISLOCATIONS OF THE HEAD OF THE HUMERUS, PRACTISED AT THE BRISTOL INFIRMARY.

This is detailed by Mr. W. F. Morgan, late house-surgeon to the institution.

"As soon as the patient is seen, and the nature of his injury ascertained, he is placed sideways on a common chair, with the dislocated arm hanging over its back, which is padded for the reception and support of the axilla. A reel-towel is then fastened on the arm, immediately above the condyles, by a running noose, with the knot outwards; and its lower part is formed into a loop some way above the ground, to receive the foot of the surgeon. One or two assistants press on the shoulder, much in the same way as Mr. Toogood advises, to fix the scapula and prevent the patient from rising, and the surgeon puts his foot, or, occasionally, his knee, into the loop of the towel, and gradually and unremittingly bears on it with the whole weight of his body. In a very little time, generally within three or four minutes, the head of the bone slips into its place with an audible jerk; the patient's sufferings are moderate, and of short duration, and the use of his arm is soon restored.

In dislocations of long standing, of course other and more powerful means are requisite; but, in several of the cases to which I have alluded, many hours, and even days had elapsed, before the patients applied for relief. I have, likewise, notes of the case of a strong and muscular man, who was admitted with a dislocation of the shoulder, complicated with fracture of the humerus at its middle, in whom the reduction was effected as readily as usual, by binding up the arm with splints, and applying the towel on the outside of them."

In the great majority of recent cases, the reduction is readily, simply, and quietly effected by the heel of the surgeon in the axilla. No towels are necessary—no further preparation is required than removal of the surgeon's boot—no other requisite than a moderately-clean pair of stockings.

We now take leave of this volume of Transactions for the present. There are many articles for notice—some for criticism, in our next. Till that makes its appearance, we wish the contributors adieu. It would appear ill-natured to hint a defect on parting, yet censorious readers might complain of prolixity in several of the articles.

---

### ON THE CERTAINTY AND SAFETY WITH WHICH THE OPERATION FOR THE EXTRACTION OF A CATARACT FROM THE HUMAN EYE MAY BE PERFORMED, AND ON THE MEANS BY WHICH IT IS TO BE ACCOMPLISHED. By G. J. GUTHRIE, F.R.S. &c. Octavo, stitched, pp. 47, price 2s. 6d. London, 1834.

This pamphlet is printed for the benefit of the Ophthalmic Hospital, a valuable institution, much wanted and much prized at the western end of the metropolis, and which owes its maturity, if not its birth, to Mr. Guthrie. We have so frequently introduced this able surgeon to the notice of our readers, that we may proceed, without farther preface, to extract from the pages of the work before us a few of the practical recommendations contained in it.

Mr. Guthrie alludes to Wenzell's remark, that an oculist must spoil a hatful of eyes before he can extract a cataract well. This Mr. G. believes must be the case, if the surgeon implicitly pursues the directions which were given at that period. We can well believe that Mr. Guthrie's hatful is a small one—a low-crowned modern beaver, not admitting of comparison with the huge and capacious structures which formerly surmounted the human head.

We shall endeavour to present Mr. Guthrie's sentiments on some of the points which have excited most discussion amongst oculists, as well as upon those in which his experience may render his opinion valuable.

The following are his directions respecting the manner in which the patient should be prepared for the operation.

" It appears to me, that the same preparation only is required that would be considered necessary previously to the performance of any surgical operation of importance, and particularly about the head. If the patient is in good health, and free from any appearance of disease, nothing more is wanting than a couple of gentle doses of physic, and moderate abstinence during the week preceding the operation. If the patient has lived regularly well, and is disposed to be of a full habit, twelve ounces of blood may be taken from the arm the evening before the operation. If the health is impaired from an irregular mode of life, it should be amended. If a fit of the gout is impending, or is supposed likely to occur, it should be allowed to pass over before the operation is performed; or if the patient has a strong determination of blood to the head, he should be cupped a few days before it is done, in addition to the bleeding recommended on the evening preceding the operation; and the same or greater precautions should be taken when there are indications of a tendency towards paralysis or apoplexy. These are, however, only the ordinary precautions which a well-informed surgeon would take in all other cases. When the operation is to be done on nervous persons, care must be taken on the other hand to prevent any undue depression of strength or spirits."

Mr. Guthrie was formerly of opinion that both eyes should not be operated on at once. He was not so certain of success then as now, and he does recommend the simultaneous peformance of the operations, chiefly for the following reasons :—If an accident should occur, and a very vigorous or prolonged after-treatment be necessary, the patient, whatever may be the result, will not readily submit to a second; and, if an elderly person, will not be in a state to submit for a great length of time afterwards; and if that second operation should be unlucky, requiring also a vigorous after-treatment, the loss of health, and ultimately that of life, may be the consequence. Elderly persons can generally bear a vigorous treatment *once* well, and recover from it completely; but they cannot often bear and recover from it *twice*.

The operation of extraction should always be performed with the corneal incision in the direction upwards. Mr. Guthrie's directions for the management of the lids and of the eye are most judicious.

" For the operation on the right eye, the surgeon should place himself behind the patient, and he will usually find it necessary to stand on a stool, in order to raise himself to such a height, that he may readily lean over, and have his hands at perfect ease; and in that position and distance from his own head or chest, which is most convenient to him. The patient's head being a little inclined backwards, and duly, although gently and comfortably supported by the cushion, or back of the chair; the surgeon leaning over from behind, brings the *two* fore-fingers of the left hand over the forehead gently down on the eyelid, and raises it up slowly and tenderly, so as to fix it ultimately against the upper edge of the orbit; and to be able to retain it there so perfectly with the end of *the*

fore-finger only, that the patient cannot lower it or close the eyelid by any effort he can make. He should also be able to do this and to make a little pressure on the eyeball, in order to fix it at the moment the incision is begun. As soon as the index finger is in this position, the second finger leaves the upper, and lowers the under lid, and fixes it against the edge of the orbit below. The eye is thus completely exposed,. and may be almost fixed between the two fingers. To do all this well, requires a certain degree of practice, but which is very easily acquired. It must be done very gently, very tenderly, and without giving pain, or almost uneasiness. The error usually committed, is in using too much force with the extremity of the finger, which gives pain, and makes the patient swerve; and it is an error of such great importance, that the surgeon must practise this part of the operation until he feels that he does it as a matter of art, not of force.

The left eye may be opened, and fixed in a similar manner; or the surgeon standing before the patient, raises the upper lid with the side of the fore-finger of the left hand, and depresses the under lid with the thumb, the hand being over the nose. The pressure of the fore-finger tends to fix the eye at the same time, and to render it as immoveable as possible, and this mode of proceeding I generally adopt in preference, for the left eye."

Mr. Guthrie ably exposes the fallacy of the popular belief, that the eye is a very delicate and a very sensitive organ, and hints that the notion has been propagated and fostered for interested purposes. He observes that such a notion cannot long be entertained, by any who are in the habit of witnessing the performance of operations on the organ. Mr. G. observes, that full exposure of the eye diminishes, in a great degree, its sensibility. He recommends the surgeon to acquire the power of operating, without the assistance of another in steadying the eye; unity of purpose is gained by this. The knife which he prefers is spear-pointed, and blunt on the back.

"The width depends on the maker, my rule being that it shall be as broad as the perfection of the point will admit, and the artist finds that he cannot put the best point on a broad instrument, so that it is essentially rather a narrow knife. It should be of that degree of equal thickness which will admit of its passing through the cornea with the greatest facility, and of its bearing the very best possible point and edge. Every thing in this operation depends on the point of the knife, for the operation must fail either partially or totally if the point of the knife is not a good one; nothing else signifies so materially, and it must be attended to. The surgeon should never depend on the instrument maker: he should try it on the fine piece of thin leather which ought to have a place in every case of eye instruments. Through this it should pass as through water, without the slightest sound; for if the slightest sound be heard, the knife is unfit for use. Held between the fore-finger and thumb, with the little finger resting outside the orbit, the surgeon awaits calmly the steadying of the eye, which should be fixed nearly in the centre, the pupil looking directly forwards. The flat part of the cornea-knife may now be made to touch the face of the cornea as the probe did before, in order to ascertain that the patient is totally free from alarm, and that the eye is steady."

Mr. G. sides with the advocates for the use of belladonna before the operation. It should be applied on the previous evening. After stating that the upper half of the cornea must be divided, Mr. Guthrie makes some lively remarks on the mode in which the knife is to be made to enter it.

"The manner of entering the point of the knife is disputed. The cornea being composed of several layers, constituting a substance of a certain thickness, there is some danger of passing the knife between the layers, and not across the anterior chamber of the eye in front of the iris, if care be not taken that the cornea is fairly penetrated in the very first entering of the knife; which accident may happen, if the anterior chamber is small, and the iris is close to the cornea. In order to avoid this error, modern authors (I believe, without an exception), recommend that the knife should be entered in the direction of the iris, as if the point were to be carried directly against it, but that as soon as the cornea is penetrated, and the point is in the anterior chamber, the handle of the knife is to undergo a sort of almost imperceptible depression towards the temple, by means of which, the blade is to be placed with its flat surfaces parallel to the iris, across the anterior chamber; and it is said that the more quickly this is done, the less chance is there of the escape of the aqueous humour. To all this I object in the most decided manner; the gentlemen who think they do as they say, are, I believe, mistaken; and when they do, they do nothing but mischief. The very turning, or attempting to turn the knife, generally leads to the escape of the aqueous humour, the very thing they want to preserve, and to all the evils it is most desirable to avoid. The knife should, in my opinion, be held flat, and the point is to be introduced steadily in the same direction at the proper place; and if the operator can neither see, nor feel when it has penetrated the cornea, and is in the anterior chamber, the sooner he abandons operating the better. If a man has eyes, he can see when the point of the knife has entered the anterior chamber; and if he has fingers, he will feel when the resistance offered by the cornea is overcome. The young surgeon, instead of practising these manœuvres, which are worse than useless, should practise on sheeps' eyes, until he has acquired that tact, which will enable him to know when his knife is in the right place."

Mr. Guthrie alludes, in a forcible manner, to the disposition in the eye to turn inwards at the moment of the incision, and enjoins the utmost coolness on the part of the operator. He dwells very strongly on the fact that the inner layer of the cornea is more tough than the outer, and gives many interesting practical hints to the operator. We regret that we cannot introduce them. For the following piece of information and advice, we must certainly make room.

"When the perforation or penetration is completed, and well done, the patient and surgeon are in a very different relative situation to each other; before it was done, the eye was almost entirely under the control of the patient; after it is done, the eye is almost entirely under the control of the surgeon. It moves with, and of course follows the motions of the knife, and if it has turned inwards, even into the very internal angle, the knife can bring it gently back to its due central position, but not without the loss of the whole of the aqueous humour; and the iris is now seen bulging forwards on the edge of the knife, sometimes even overlapping it. It is at this point that a young operator is confounded, he sees that the iris must be cut, if it cannot be moved out of the way; he thinks he has failed, becomes confused, hesitates, and does mischief, or withdraws the knife and abandons his operation: or if he is a clearer-headed person, he bethinks him of the directions he has read upon this subject, and proceeds accordingly; but he does not succeed one whit the better, and in despair, accuses himself of awkwardness. But he is not, however, the person in error—it is those who wrote the directions. I do not mean to say that these gentlemen have willingly deceived the public, but I do say it is my opinion, that they have told only half the truth. It may be that they did not know the other half, and I have no objection to its being so considered, except that I shall be set down for a discoverer of it, whether I will or not, which I am very desirous to avoid. The directions given in such a case by the Baron de Wenzel, and which the late Mr. Ware said, were the most important in his whole book are, that the cornea must be gently rubbed with the point of the fore-finger, which causes a contraction of the pupil and consequent drawing back of the iris, when the surgeon must complete the operation; but if the iris again fall forward, before this is accomplished, he must keep the finger on the cornea, until it is effected, by which, all danger of the iris suddenly protruding will be avoided. It is also recommended that the surgeon should wait a little, and allow the spasm to subside; and he may wait and rub, and wait again, and then rub again if he pleases, but he will rarely succeed, unless he does something more, and that is, to raise the eye, or in other words, to draw it as it were out from the orbit, whilst at the same time he presses the cornea flat against the blade of the knife. This is the other and the best half of the secret, and without he does which, he will not succeed in disentangling the iris. A little consideration will, I think, show why it can only be done in this manner. When the aqueous humour has escaped, the cornea becomes flaccid, and the remaining humours of the eye advance, or are brought forward by the action of the recti muscles, so as to press the iris against it. If the knife is between the iris and the cornea, it keeps these parts asunder, as far as its width extends, but not farther, and as it raises the cornea in every part, it would make a vacuum below its edge, between the iris and the cornea, if the iris did not rise up to fill the space, or if the air did not rush in to do it. This effect of the air, is however counteracted by the muscles acting on the eye with greater power; and the consequence is, that the iris is forced upwards into the vacant space, and the air, if any has entered, is expelled. The iris can, however, be only elevated or protruded to a certain extent, in consequence of its circular attachment, and of its disposition to contract towards its pupillary edge, or centre. When the cornea is well raised, so as in some degree to raise the eye along with it, whilst, at the same time, the cornea is pressed against the blade, the iris slides from between them, and the operation may be completed, so as to give rise to a highly-successful operation. There is now another secret to be disclosed, of yet greater importance to the young operator, it is, that the effect of an injury to the iris is very greatly overrated, and that if the operation cannot be completed without injuring it, the injury must be committed. The real truth is, that *no good operators* in this great city of London do otherwise. When the iris bulges much over the edge of the knife, it is often not possible to get it quite clear, by any effort, exertion, or dexterity, on the part of the operator. If any man says otherwise, I do not hesitate to say that he is in error, and that his own operations, if he has ever done any, will show it. This being the case, the operator has only a choice of evils, to proceed under any circumstances, or to abandon the operation. This I have done, and the consequence was, that as much inflammation followed as if the operation had been duly performed; and the eye was not upon the whole in a very favourable state for a future operation, the iris being usually a little injured, and the consequent inflammation rendering the pupil less regular and dilateable than it ought to be. If the operation is on the contrary completed, the iris is slightly shaved as the knife advances, or a piece may even be cut out, but the patient will, nevertheless, have a very good eye. The cut in the iris will often not be discernible, unless the upper lid is raised to look for it, and he will see remarkably well, after a very speedy recovery. In fine, I may say the operation is always done in this manner by all those who know what they are about, and the eyes of persons of all ranks who have been operated upon, either in London or on the Continent, prove it. It is possible that the operators may not know what they have done, or how they did it; they may have supposed they were doing one thing, while they were actually doing another. I do not dispute this, or anything else, in the least, but I write to give such information to young surgeons as will enable them to operate successfully, and to know why and wherefore it is that they are successful."

For many valuable observations, and for some particulars respecting a patient on whom De Graefe operated at Mr. Guthrie's request, whilst Mr. G. himself extracted the lens of another, we must refer the curious reader to the pamphlet itself.

There is a circumstance attending the protrusion of the vitreous humour with which young operators should be familiar.

"The lens," says our author, "does not however always come out so easily and regularly. It sometimes happens that instead of rising up by its upper edge, the vitreous humour, which appears black because it is transparent, and allows the black pigment beneath to be seen through, is perceived pushing forwards between it and the iris. Nothing can prevent a portion of this vitreous humour being protruded or expelled, and no attention need be paid in order to obviate it, for it cannot be done. But attention must be paid to the fact, that its expulsion under pressure will not in general be accompanied by that of the lens, which, having lost its support, will sink down towards the bottom of the eye, and must infallibly cause its destruction by inflammation, if not removed. The surgeon aware of this circumstance, and knowing that pressure of any kind, that even the mere action of the muscles, will cause the expulsion of the vitreous humour without the lens, passes either the large or the small hook under the edge of the lens, through the vitreous humour, which begins to escape when its enclosing membrane is pierced by the hook, the point of which is to be fairly stuck into the under part of the lens, and thus drawn out. A portion of vitreous humour must of course escape, it cannot be prevented, it was inevitable from the first; but the great object, the extraction of the lens, has been attained. If the surgeon hesitates, and does not calmly and steadily introduce his instrument, and hook the lens at once, the vitreous humour begins to escape, the lens sinks, and the eye will be lost, if he does not instantly pass the hook through the pupil, and hook the lens, as he would catch a fish with a landing hook. There is no alternative, it must be done, or the eye will be destroyed."

Mr. Guthrie observes, that the loss of a portion of the vitreous humour has been a surgical bug-bear in the operation. It is an evil, but not a very great one. The principal inconvenience accruing from it is an irregularity of the pupil, which does not in many instances impair vision. If a very small portion of vitreous humour be lost, it is usually followed by severe inflammation; if a large quantity has escaped, the inflammation is usually less. The pupil is apt to be drawn in the direction in which the expulsion or evacuation has occurred, and usually retains more or less of that distortion. The best method of causing its return to its natural place, is to allow the lid to fall, and then to rub it very gently with a soft and wet sponge, for two or three minutes, which often brings it very nearly back to its central position. Another and a greater inconvenience arising from the evacuation of the vitreous humour, is, that a portion interposes between the cut edges of the cornea when they are supposed to be placed in apposition; and whilst it prevents a rapid union between these parts, it also tends, by allowing the newly-secreted aqueous humour to escape, to draw the iris into the line of the incision. If this takes place, the iris adheres to the cut surface, the pupil is permanently and more completely drawn in that direction, and the anterior chamber of the aqueous humour is less perfect, both in shape and appearance, in consequence of the cornea and the iris being brought nearer to each other. This inconvenience arising from the interposition of the vitreous humour between the edges of the incision in the cornea, is to be avoided, as far as it can be done, by carefully removing with the curette any part of it which can be seen, and by gently rubbing the eye in the way directed, in order to cause the pupil to return to its proper place.

The adjustment of the cornea is of course a matter of importance. The eye should appear flat. If it does not the improper fulness arises from some displacement of the vitreous humour, which has not burst the membrane in which it is inclosed, but has been pushed forwards by the action of the muscles of the eye, so as to cause the iris to project; the flap of the cornea is seen lying upon it, and is separated by it from the other edge, with which it ought to be in apposition. Adhesions of the iris and many other evils will ensue unless this be prevented. The vitreous humour must be pressed back into its place by gentle friction on the eyelid covering the ball, by which it may in general be accomplished; but if it will not yield to this treatment, the hook should be introduced through the pupil, and the membrane of the vitreous humour should be punctured, so as to allow of the evacuation of one fifth of it, when the eye flattens, and the cut edges of the cornea can be brought into apposition. The extent of evil is now known, and can be duly estimated, and guarded against. In the former case it cannot; and of the two evils, the last should always be chosen, inasmuch as it will lead to the recovery of the patient with a good eye, which the other cannot do.

"The eyelid, being at last closed, is to be retained in that situation by compress and bandage; and it is not to be raised, or the eye opened, for a certain length of time, the duration of which is different, according to different writers. It ought to depend on circumstances; the earliest period is on the third day, but the fourth is much better; and if any difficulties have been experienced during the operation, and there is reason to suspect that union has not taken place, the lid should not be raised for at least a week; although the lower lid may be depressed, to enable the surgeon to see the state of the ball of the eye, and to prevent the accumulation of fluids in it. I never open the eye before the fourth day, although I frequently depress and clear the lower lid on the third, and obtain a view of the globe, the whiteness of which is the best sign of the absence of inflammation. When the eyelid is raised too soon, that is before the union of the cut parts of the cornea is complete, the movement of the globe of the eye, which inevitably follows the raising of the lid, often separates the incompletely united parts; pain of a more or less acute nature is soon felt in the line of the incision, inflammation follows, ulceration succeeds and the cornea is only completely united by a slow process, attended by opacity, and generally by a drawing up of the pupil; all of which evils would have been avoided in a great measure by delaying the raising of the lid for two or three days, until the parts were more complete consolidated."

The state of the eye can be ascertained by the appearance of the upper lid, which should be carefully inspected twice daily. Swelling of the lid is a test of inflammation of the organ. On the evening of the operation Mr. Guthrie usually directs the abstraction of from 12 to 16 ounces of blood. It should never be carried so far as to occasion sickness. The eye may be occasionally fomented with a warm decoction of poppies, care being taken that the eyeball is not moved, so as to cause a separation of the divided parts; all remedies likely to cause sickness should be avoided during the course of the treatment, and active aperients for the first two or three days, will, in all probability, be unnecessary, from their having been adopted previously to the performance of the operation. Mr. Guthrie strongly deprecates opening the eye or meddling with it.

Mr. Guthrie has seen one instance of reproduction of the lens.

The practical nature of the observations contained in this pamphlet, and the candid manner in which Mr. Guthrie communicates the results of his experience to the profession, will more than excuse the length of this notice. Those who take an interest in ophthalmic surgery, (and who, at the present day, do not?) will peruse the quotations we have made with much interest.

---

RECENT ANATOMICAL AND PATHOLOGICAL PLATES.

1. PRINCIPLES AND ILLUSTRATIONS OF PATHOLOGICAL ANATOMY, &c. By *J. Hope*, M.D. F.R.S. &c. Part XI. May, 1834.
2. ILLUSTRATIONS OF THE ELEMENTARY FORMS OF DISEASE, &c. By *R. Carswell*, M.D. &c. Fasciculus V.—Softening.
3. A SERIES OF ANATOMICAL PLATES IN LITHOGRAPHY, &c. Edited by *Jones Quain*, M.D. &c. Fascic. XI. XII. and XIII.
4. ILLUSTRATIONS OF ALL THE MOST CELEBRATED MEDICAL AND SURGICAL WORKS. Numerous numbers.

THE whole of these works are continuations of a series, to which we have already drawn attention on several occasions. The two last are chiefly of an anatomical character; that of Mr. Quain is so exclusively. On these we made some particular observations in our last number. We complained of the smudginess of some of Mr. Quain's plates, and we are sorry to observe that the defect is still apparent. That gentleman is not himself the lithographist, but surely he might exercise wholesome control on the operations of his draughtsman. The plates of Mr. Quain will be as dear as Cloquet's, and, so far as we can judge by what have yet appeared, not by any means so good. We observed that we should feel great pleasure in noticing any improvement which might be effected. That pleasure, apparently, is yet to come.

The letter-press of Dr. Carswell's fasciculus has been fully considered in the Review Department of the present number. It only remains for us to offer a brief account of the plates. They equal those which have preceded them, eulogy of no mean character. The first plate contains two figures, one representing the effects of the gastric acid on the stomach—the other, perforation of the fundus of the organ. The second plate exhibits four figures. Three display softening from the action of the gastric acid—the last, pale inflammatory softening of the mucous membrane of the colon. The third plate is also occupied with four figures, the object of which is to portray red and pale inflammatory softening of the brain. The fourth plate is devoted to the delineation of softening of the brain from obliteration of the arteries. It would be difficult to select particular figures for commendation or criticism. They do credit to the skill and the industry of Dr. Carswell.

We were accidentally prevented from doing full justice to this part of the able work of Dr. Hope in our Review Department. Our notice of it here must necessarily be imperfect.

It is occupied with the description and the representation of some of the diseases of the uterus, and with those of the spleen, the bladder, and the kidneys.

There are four plates, containing thirty drawings. The outline is distinct; the colouring, which is effected by as light and as simple washes as possible, is not possessed of that gaudiness and glare, too frequently observed in pathological drawings. Waiving any further attempt at description, which would be inadequate to convey a satisfactory idea of them, we shall endeavour to select, from the descriptive portion, as much useful information as our limited space enables us to introduce.

The following is Dr. Hope's experience on the subject of the corroding ulcer of the uterus.

"Several cases," says he, "have fallen under my observation within the last few years, and the appearances presented after death were,—a dirty-greenish and brownish, sloughy and shreddy ulceration, with more or less adhesion and hypertrophy of the surrounding cellular tissue and other contiguous textures,—the products of chronic inflammation, propagated to them when the disease was far advanced. I have sometimes seen the ulceration penetrate into the bladder and rectum. On examining the corroding ulcer per vaginam, the erosion may be easily felt and its extent estimated, but no thickening or induration of the uterus can be distinguished. The latter circumstance, therefore, will materially assist in forming the diagnosis from cancer; and further assistance will be derived from the fact, that when slight pressure is made with the finger on the corroding ulcer, the patient does not suddenly shrink, as in cancer, but merely complains of a little soreness. The establishment of this diagnosis is important, because, with judicious management, the progress of the corroding ulcer may be retarded even for many years; and the practitioner is enabled to inform the patient that, if she will submit to a few privations, her sufferings will not be great."

After describing scirrhus and encephaloid disease of the uterus, Dr. Hope remarks that there is reason to believe that a slight degree of the malady may sometimes remain in an indolent state even for many years, provided the individual adopt proper regiminal precautions. Eventually, however, the constitution becomes tainted, and the disease then tends unrelentingly to its fatal termination. Any violence done to the part appears greatly to hurry on this event. A stout, plethoric, and healthy-looking woman of fifty, subject for some years to a slight uterine affection, received a kick, which brought on hæmorrhage and pain. From this time the pulse rarely fell below 110, and there were constant lancinating pains, complete anorexia, vomiting of most ingesta, and, in four or five months, great emaciation. A thickened, ulcerated, and exquisitely painful state of the os uteri can be felt *per vaginam.*

Passing over our author's observations on other affections of the uterus, we may extract his brief account of the diseases of the fallopian tubes.

"Acute inflammation seldom attacks the tubes, except in connexion with inflammation of the uterus and ovaries, already described: it may produce pus, and lead to adhesion and obliteration.

Obliteration of a part or the whole of the tubes may also arise from other causes, as chronic inflammation, congenital adhesion, an accidental membrane stopping the uterine orifice, disease of the fimbriæ, or their agglutination to the ovary or other parts.

When the two extremities of a tube are obliterated, serum or mucus may accumulate within, to the extent even of several pints. The fluid may also consist of pus."

The only other subject to which we can allude, is to the formation of cysts in the kidney—an organ remarkably prone to them. When the ureter is plugged up, the pelvis of the kidney is of course distended, the calyces opening into it are distended also, the glandular structure of the kidney is compressed, and ultimately absorbed, and at last a mere multilocular cyst may remain. Of this, most persons accustomed to the examination of dead bodies must have witnessed numerous examples.

"Cysts by *production* are formed by a different mechanism. The cortical substance of the kidney is composed of glandular granules, each of which encloses a sort of cavity or lax web. When the communication of a granule with the tubular substance is obliterated, the granule dilates by the accumulation of the liquid which it secretes, and its walls soon undergo a transformation into fibrous membrane; for distension and compression cause atrophy of the proper tissues of organs, and development of the cellular element. Thus a cyst is formed; and, as the same cause generally operates on a number of granules at once, a number of cysts are the result: accordingly, though we occasionally find only one, two, or three, it is more common to find dozens, and even hundreds, scattered throughout the cortical substance, especially at the surface, the tubular substance remaining exempt from them. Their ordinary size is from a millet seed to a nut, but they sometimes become much larger, and augment the kidney to five or six times its natural volume. The contained matter is various: sometimes a limpid serum, variously tinted; sometimes a muddy, blackish, or yellowish fluid, and occasionally even cretaceous matter,—differences no doubt corresponding, says Cruveilhier, with the numerous modifications which the vitality of the walls undergoes.

When the cysts are numerous, the intermediate renal tissue becomes compressed and atrophous, sometimes to such a degree as to be undistinguishable. A slight quantity remaining, however, will often suffice to carry on the urinary secretion.

There is also another mode in which cysts are produced in this, as in all other glandular organs of granular structure, viz., at the expense of the serous cellular tissue existing in the organ. Cysts of this kind, seldom numerous, occasionally attain an enormous size. The contained fluid is serous, and is scarcely, if at all, coagulable by heat: it may become more or less purulent by inflammation of the cysts. Mr. Cæsar Hawkins met with a cyst containing about five pints of serum, and his account of it affords the best history of the affection in the kidney that has appeared. He denominates it an *aqueous encysted tumour*, and believes it to correspond with the same tumour in the liver."

Here we must bring this short notice to a close, recommending the work of Dr. Hope to the attention of those who desire to keep pace with the pathological knowledge of the day.

